# Sulfonylimino Group Transfer Reaction Using Imino-λ^3^-iodanes with I_2_ as Catalyst Under Metal-free Conditions

**DOI:** 10.3390/molecules24050979

**Published:** 2019-03-11

**Authors:** Akira Yoshimura, Cody L. Makitalo, Melissa E. Jarvi, Michael T. Shea, Pavel S. Postnikov, Gregory T. Rohde, Viktor V. Zhdankin, Akio Saito, Mekhman S. Yusubov

**Affiliations:** 1 Research School of Chemistry and Applied Biomedical Sciences, The Tomsk Polytechnic University, 634050 Tomsk, Russia; postnikov@tpu.ru (P.S.P.); yusubov@mail.ru (M.S.Y.); 2 Department of Chemistry and Biochemistry, University of Minnesota Duluth, Duluth, MN 55812, USA; makit003@d.umn.edu (C.L.M.); jarvi201@d.umn.edu (M.E.J.); sheax152@d.umn.edu (M.T.S.); vzhdanki@d.umn.edu (V.V.Z.); 3 Marshall School, Duluth, MN 55811, USA; greg.rohde@marshallschool.org; 4 Division of Applied Chemistry, Institute of Engineering, Tokyo University of Agriculture and Technology, 2-24-16 Naka-cho, Koganei, Tokyo 184-8588, Japan; akio-sai@cc.tuat.ac.jp

**Keywords:** iminoiodanes, imination, sulfide, sulfilimine, phosphine, iminophosphorane, amidyl radical, catalytic cycle, iodine

## Abstract

A new practical procedure of imination for sulfide has been developed. The treatment of (*N*-tosylimino)-phenyl-λ^3^-iodane, PhINTs, with various sulfides in the presence of a catalytic amount of I_2_ under metal-free conditions affords the corresponding *N*-tosylsulfilimine compounds with moderate to good yields. This facile transfer procedure of the sulfonylimino group can also be applied to triphenylphosphine to produce the respective iminotriphenylphosphoranes in high yields. According to the reaction mechanism studies, the process of imination from (*N*-tosylimino)-phenyl-λ^3^-iodane to sulfide under the conditions may involve radical steps within the reaction mechanism.

## 1. Introduction

Sulfur–carbon ylides and sulfur–heteroatom ylide compounds are widely used in many chemical reactions as important transformational reagents [1,2,3,4,5]. Numerous reactions employing sulfide ylides have been demonstrated in organic synthesis. In particular, sulfur–nitrogen ylides [6,7,8], *N*-sulfonyl sulfilimines, such as mono-aza analogues of sulfoxides, are common aziridination reagents [9], epoxidation reagents [10], or palladium metal ligands [11]. Sulfilimine compounds are also used as efficient synthons for sulfoximines, which can be easily prepared by the appropriate oxidants, and these compounds are used in medicinal or synthetic chemistry [12,13,14,15]. Synthetic methodologies for directly introducing *N*-sulfonylimino groups to sulfide atom using oxidants with nitrogen source have been developed [16,17,18,19].

**Scheme 1:**
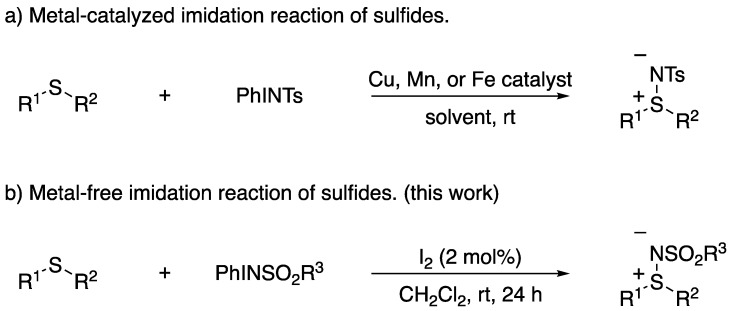
Transfer reaction of sulfonylimino groups from imino-λ^3^-iodane to sulfide atom. (**a**) Metal-catalyzed imidation reaction of sulfides, (**b**) Metal-free imidation reaction of sulfides.

Organohypervalent iodine compounds are known as efficient oxidative reagents for organic synthesis because of the exceptionally high leaving ability of the iodobenzene group [20,21,22,23,24]. The versatile reactivity of hypervalent iodine(III) compounds allows for various bond formations, some of which are otherwise difficult reactions in the absence of iodine(III) reagents. Particularly, *N*-sulfonylimino-λ^3^-iodanes represent an important class of hypervalent iodine(III) compounds, which are commonly used as *N*-sulfonylimino group sources or selective oxidative reagents for various organic substrates [25,26,27]. For example, the reaction of sulfides with (*N*-tosylimino)-phenyl-λ^3^-iodane in the presence of Cu, Mn, or Fe metal catalyst gave the corresponding sulfoximine compounds (Scheme 1a) [28,29,30,31,32,33]. However, a metal-free condition for reactions is a significant goal in the development of eco-friendly reaction methodology. Recently, Lamar and Nicholas reported the C-H amidation reaction of saturated or unsaturated hydrocarbons using imino-λ^3^-iodanes in the presence of a catalytic amount of elemental iodine under transition metal-free conditions [34]. Lamar’s group also reported the imidation reaction of aldehydes under similar reaction conditions [35]. Metal-free aziridination reactions of styrenes using (*N*-tosylimino)-phenyl-λ^3^-iodane in the presence of I_2_-tetrabutylammonium iodide combination have been developed by Minakata and co-worker [36]. To the best of our knowledge, however, I_2_ mediated metal-free imination reactions of sulfides using imino-λ^3^-iodanes have not been developed. In this paper, we report the sulfonylimino group transfer reaction from imino-λ^3^-iodane to sulfide atom under metal-free conditions (Scheme 1b). Reaction mechanisms of this sulfonylimino group transfer reaction may involve the radical process under reaction conditions.

## 2. Results

### 2.1. Optimization of Reaction Conditions

Our approach to metal-free sulfonylimino group transfer reaction is based on previously reported reaction conditions such as (*N*-tosylimino)-phenyl-λ^3^-iodane in the presence of I_2_ as a catalyst [34,36]. In the initial experiment, we investigated the reaction of thioanisole **1a** (1 equiv.) with (*N*-tosylimino)-phenyl-λ^3^-iodane **2a** (1.2 equiv.) in the presence of a catalytic amount of both I_2_ and tetrabutylammonium iodide (TBAI) as an additive under various solvents at room temperature (Table 1, entries 1–9). We have found that dichloromethane is an efficient solvent for the metal-free imination reaction of thioanisole **1a** using (*N*-tosylimino)-phenyl-λ^3^-iodane **2a**. The presence of TBAI as an additive did not affect this reaction (entry 10). However, in the absence of I_2_ as a catalyst, the reaction dramatically changed, resulting in a low yield of the formation of the desired *N*-tosyl sulfilimine **3a** (entries 11 and 12). Extension of the reaction time was effective for the desired product **3a** yield (entry 14). Decreasing the amount of I_2_ catalyst from 10 mol% to 2 mol% increased the yield of *N*-tosyl sulfilimine **3a** (entries 14–16). These results implied that the slow generation of the activated species was very important for this reaction because the species generated from (*N*-tosylimino)-phenyl-λ^3^-iodane **2a** and I_2_ were probably unstable in the reaction mixtures. Meanwhile, further reduction of the amount of I_2_ catalyst and a longer reaction time led to a decreased amount of the desired product **3a** (entries 17 and 18). Finally, performing the reaction under the dark conditions slightly suppressed the product yield, which supports a radical pathway in the reaction mixtures (entry 19) [36].

### 2.2. Scope of Reactions

According to the optimized conditions with 2 mol% I_2_, we have investigated the conversion of various substituted sulfides **1** to the corresponding sulfilimines **3**. In general, the reactions of thioanisoles **1a–i** with either electron-donating or electron-withdrawing substituents using (*N*-tosylimino)-phenyl-λ^3^-iodane **2a** in the presence of I_2_ as the catalyst gave the corresponding *N*-tosyl sulfilimine compounds **3a-i** in moderate to good yields (Table 2, entries 1–9). In the reaction of the sterically hindered *o*-chlorothioanisole **1f** or the strong electron-withdrawing substituted *p*-nitrothioanisole **1i**, products **3** were obtained in 59–69% yields (entries 6 and 9). The reaction of diphenylsulfide **1j**, benzyl phenyl sulfide **1k**, and dibenzyl sulfide **1l** gave the respective products **3j–k** in moderate yields (entries 10–12). This reaction also worked for the acyclic **1** or cyclic aliphatic sulfides **1m-p** to give the desired sulfilimines **3m-p** in good yields (entries 13–16). However, the reaction with the most steric bulky sulfide, *di*-tert-butyl sulfide **1q**, afforded the desired product **3q** in only 8% (entry 17). As expected, the reaction of **1a** using imino-λ^3^-iodanes **2b–d** with different substituents afforded the corresponding sulfilimines **3r–s** in moderate to good yields (entries 18–20). This metal-free procedure gave comparable or higher yields of sulfilimines compared to the previously reported metal catalyst mediated *N*-tosyl sulfonylimino group transfer reaction to sulfide from (*N*-tosylimino)-phenyl-λ^3^-iodane [28,29,30,31,32,33]. Particularly, the scaled up reaction of **1a** (1 mmol) under optimized conditions gave the desired product **3a** in 76%.

**Scheme 2:**
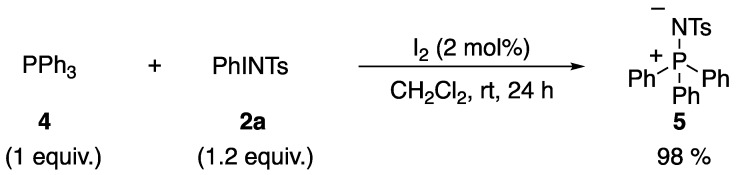
Transfer reaction of *N*-tosyl sulfonylimino groups to triphenylphosphine **4**.

Moreover, the reaction of triphenylphosphine **4** with (*N*-tosylimino)-phenyl-λ^3^-iodane **2a** under optimized conditions also allowed the transfer reaction of *N*-tosyl sulfonylimino group to give the (*N*-tosylimino)-triphenylphosphorane **5** in excellent yield (Scheme 2). The structure of **5** was confirmed by a single crystal X-ray crystallography (see Supplementary Materials) [37]. Compared to the previously reported preparation of **5** using imino-λ^3^-iodanes, our reaction proceeds under very mild conditions and affords the product in higher yields [38,39].

## 3. Discussion

### 3.1. Mechanistic Study

**Scheme 3:**
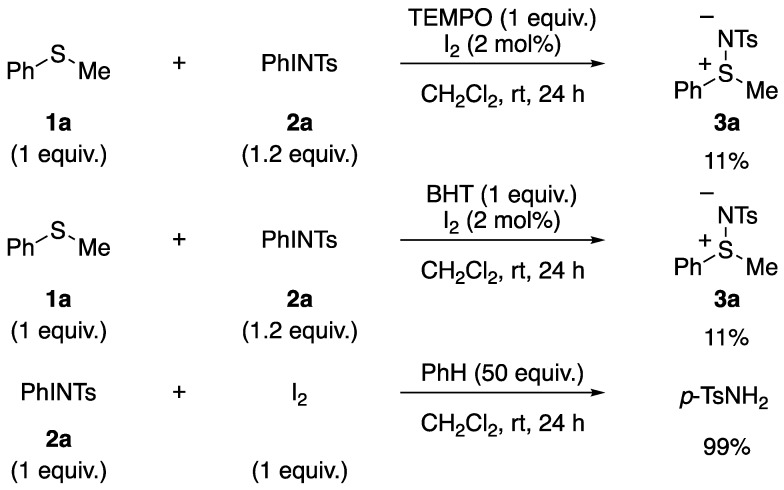
Blank experiments of imination reaction.

To clarify the mechanism of metal-free sulfonylimino group transfer reaction, we have performed several blank experiments (Scheme 3). Based on the previously reported experiment, a amidyl radical precursor like *N*,*N*-diiodotosylamide or a related species might be generated from (*N*-tosylimino)-phenyl-λ^3^-iodane **2a** with I_2_ [34,35,36,40]. A radical mechanism is plausible because performing the reaction under dark conditions results in relatively low yields of product **3a** (Table 1, entry 20). Therefore, the presence of a radical scavenger such as TEMPO or BHT in the reaction would be effective for identification of the reaction mechanism. In the case of both reactions, the desired sulfilimine **3a** was detected in the reaction mixtures in low yields around 11% as compared to 88% without the radical scavenger, which implies that the amidyl radical species were involved under the reaction mixture. When benzene was added to the reaction instead of thioanisole **1a**, azepine or *N*-phenyl-*p*-toluenesulfonamide was not detected and only *p*-toluenesulonamide was recovered from the reaction mixtures. This result suggested that highly active nitrene species were not present under the reaction conditions.

### 3.2. Proposed Reaction Mechanism

**Scheme 4:**
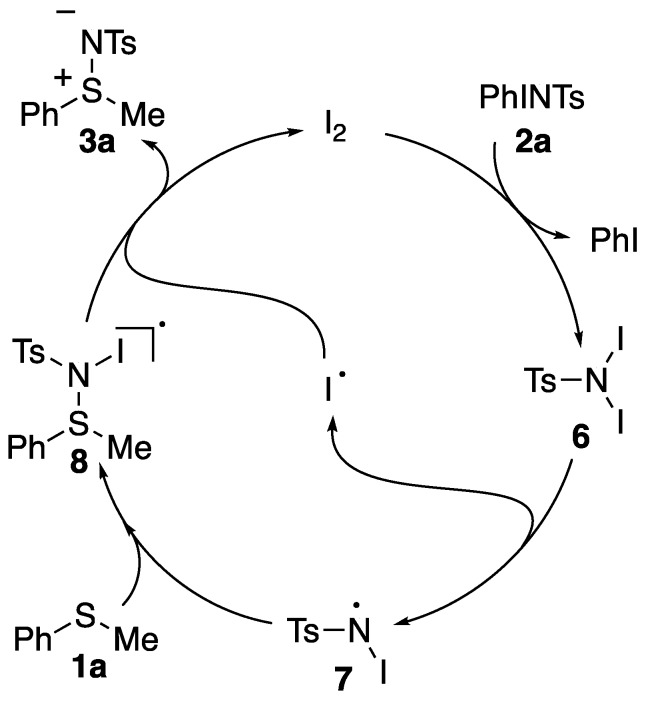
Proposed reaction mechanism.

From blank experiments and previously related results involving the amidyl radical species from imino-λ^3^-iodane **2a** with I_2_ [34,35,36,40], we proposed the reaction mechanism of imination (Scheme 4). Initially, (*N*-tosylimino)-phenyl-λ^3^-iodane **2a** reacted with I_2_ to produce *N*,*N*-diiodotosylamide **6** (or related species) followed by generation of the amidyl radical **7** and iodine radical under the reaction conditions. The generated amidyl radical **7** was trapped by thioanisole **1a** to afford the intermediate compound **8**, followed by loss of iodine radical to give the desired sulfilimine product **3a** and reproduced I_2_. The regenerated I_2_ would continue the next catalytic cycle.

## 4. Materials and Methods

### 4.1. General Experimental Remarks

All reactions were performed under dry argon atmosphere with flame-dried glassware. All commercial reagents were ACS reagent grade and used without further purification. Dichloromethane was distilled from CaH_2_ immediately prior to use. Melting points were determined in an open capillary tube with a Mel-temp II melting point apparatus. Infrared spectra were recorded on a Perkin-Elmer 1600 series FT-IR spectrophotometer, and peaks were reported in reciprocal centimeters (cm^–1^). ^1^H NMR spectra were recorded on a Varian Inova 500 and 300 MHz NMR spectrometer; ^13^C NMR spectra were recorded on Varian Inova 500 and Varian 300 MHz NMR spectrometers at 125 and 75 MHz, respectively. Chemical shifts are reported in parts per million (ppm). ^1^H and ^13^C chemical shifts are referenced relative to tetramethylsilane. X-ray crystal analysis was performed by Rigaku RAPID II XRD Image Plate using graphite-monochromated MoKα radiation (λ = 0.71073 Å) at 173 K. (*N*-*p*-Tosylimino)phenyl-λ^3^-iodane **2a** [41], (*N*-*p*-nosylimino)phenyl-λ^3^-iodane **2b** [42], (*N*-*o*-nosylimino)phenyl-λ^3^-iodane **2c** [43], and *N*-(phenylsulfonylimino)-phenyl-λ^3^-iodane **2d** [44] were prepared according to the reported procedures.

### 4.2. General Procedure for Imination of Sulfides 1 with Imino-phenyl-λ^3^-iodane 2 in the Presence of I_2_

Imino-λ^3^-iodane **2** (0.12–0.24 mmol) was added at room temperature to a stirred mixture of sulfide **1** (0.10–0.20 mmol) and I_2_ (0.002–0.004 mmol) in dichloromethane (1.0–2.0 mL). The reaction was stirred at room temperature for 24 h. After the reaction, 5% aqueous Na_2_S_2_O_3_ (2.5–5.0 mL) was added to the mixture and the solution was extracted with ethyl acetate. The organic layer was dried over anhydrous Na_2_SO_4_ and concentrated under reduced pressure. The residue was separated by column chromatography using the Hexane-EtOAc (1:1 to 0:100) to afford the pure product **3**.

*S-Methyl-S-phenyl-N-p-tosyl-sulfilimine* (**3a**) [45]. Reaction of thioanisole **1a** (25 mg, 0.20 mmol) according to the general procedure afforded 52 mg (88%) of product 5a, isolated as a white solid: mp 129–130 °C (lit. [45]; mp 131.5–132 °C); ^1^H NMR (500 MHz, CDCl_3_): δ 7.73 (d, *J* = 8.5 Hz, 2H), 7.69 (d, *J* = 8.0 Hz, 2H), 7.56–7.46 (m, 3H), 7.16 (d, *J* = 8.5 Hz, 2H), 2.84 (s, 3H), 2.37 (s, 3H).

*S-Methyl-S-(4-tolyl)-N-p-tosyl-sulfilimine* (**3b**) [29]. Reaction of methyl(4-tolyl)sulfide **1b** (28 mg, 0.20 mmol) according to the general procedure afforded 44 mg (72%) of product **3b**, isolated as a light brown oil; ^1^H NMR (500 MHz, CDCl_3_): δ 7.72 (d, *J* = 8.3 Hz, 2H), 7.57 (d, *J* = 8.5 Hz, 2H), 7.28 (d, *J* = 8.3 Hz, 2H), 7.16 (d, *J* = 8.5 Hz, 2H), 2.82 (s, 3H), 2.39 (s, 3H), 2.35 (s, 3H).

*S-Methyl-S-(4-methoxyphenyl)-N-p-tosyl-sulfilimine* (**3c**) [46]. Reaction of 4-methoxyphenyl(methyl)sulfide **1c** (31 mg, 0.20 mmol) according to the general procedure afforded 51 mg (78%) of product **3c**, isolated as a white solid: mp 147 °C (lit. [46]; mp 146–147 °C); ^1^H NMR (500 MHz, CDCl_3_): δ 7.68 (d, *J* = 7.3 Hz, 2H), 7.59 (d, *J* = 8.3 Hz, 2H), 7.13 (d, *J* = 7.3 Hz, 2H), 6.94 (d, *J* = 8.3 Hz, 2H), 3.81 (s, 3H), 2.80 (s, 3H), 2.33 (s, 3H).

*S-Methyl-S-(4-chlorophenyl)-N-p-tosyl-sulfilimine* (**3d**) [46]. Reaction of 4-chlorophenyl(methyl)sulfide **1d** (32 mg, 0.20 mmol) according to the general procedure afforded 52 mg (79%) of product **3d**, isolated as a light yellow solid: mp 111–112 °C (lit. [46]; mp 112–113 °C); ^1^H NMR (500 MHz, CDCl_3_): δ 7.71 (d, *J* = 8.0 Hz, 2H), 7.64 (d, *J* = 8.5 Hz, 2H), 7.45 (d, *J* = 8.5 Hz, 2H), 7.17 (d, *J* = 8.0 Hz, 2H), 2.84 (s, 3H), 2.36 (s, 3H).

*S-Methyl-S-(3-chlorophenyl)-N-p-tosyl-sulfilimine* (**3e**) [47]. Reaction of 3-chlorophenyl(methyl)sulfide **1e** (32 mg, 0.20 mmol) according to the general procedure afforded 53 mg (80%) of product **3e**, isolated as a white solid: mp 137–138 °C; ^1^H NMR (500 MHz, CDCl_3_): δ 7.68 (d, *J* = 8.0 Hz, 2H), 7.61 (s, 1H), 7.53 (d, *J* = 7.8 Hz, 1H), 7.44 (d, *J* = 7.8 Hz, 1H), 7.39 (t, *J* = 7.8 Hz, 1H), 7.15 (d, *J* = 8.0 Hz, 2H), 2.83 (s, 3H), 2.33 (s, 3H).

*S-Methyl-S-(2-chlorophenyl)-N-p-tosyl-sulfilimine* (**3f**) [48]. Reaction of 2-chlorophenyl(methyl)sulfide **1f** (32 mg, 0.20 mmol) according to the general procedure afforded 45 mg (68%) of product **3f**, isolated as a white solid: mp 149–150 °C; ^1^H NMR (500 MHz, CDCl_3_): δ 8.12 (d, *J* = 7.5 Hz, 1H), 7.71 (d, *J* = 8.3 Hz, 2H), 7.52–7.41 (m, 3H), 7.20 (d, *J* = 8.3 Hz, 2H), 2.87 (s, 3H), 2.36 (s, 3H).

*S-Methyl-S-(4-bromophenyl)-N-p-tosyl-sulfilimine* (**3g**) [49]. Reaction of 4-bromophenyl(methyl)sulfide **1g** (41 mg, 0.20 mmol) according to the general procedure afforded 52 mg (70%) of product **3g**, isolated as a white solid: mp 110–111 °C; ^1^H NMR (500 MHz, CDCl_3_): δ 7.70 (d, *J* = 8.0 Hz, 2H), 7.60 (d, *J* = 9.0 Hz, 2H), 7.55 (d, *J* = 9.0 Hz, 2H), 7.16 (d, *J* = 8.0 Hz, 2H), 2.83 (s, 3H), 2.35 (s, 3H).

*S-Methyl- S-(4-cyanophenyl)-N-p-tosyl-sulfilimine* (**3h**). Reaction of 4-cyanophenyl(methyl)sulfide **1h** (30 mg, 0.20 mmol) according to the general procedure afforded 55 mg (86%) of product **3h**, isolated as a light brown solid: mp 159–160 °C; IR (neat) cm^−1^: 3096, 3035, 2929, 2856, 2234, 1400, 1144, 758; ^1^H NMR (500 MHz, CDCl_3_): δ 7.83 (d, *J* = 8.5 Hz, 2H), 7.74 (d, *J* = 8.5 Hz, 2H), 7.69 (d, *J* = 8.3 Hz, 2H), 7.17 (d, *J* = 8.3 Hz, 2H), 2.87 (s, 3H), 2.35 (s, 3H); ^13^C NMR (75 MHz, CDCl_3_): δ 142.3, 141.6, 140.7, 133.5, 129.4, 126.6, 126.2, 117.1, 116.1, 38.8, 21.4; HRMS (ESI-TOF-positive mode): calcd for C_15_H_14_N_2_NaO_2_S_2_ ([M + Na])^+^: 341.0394, found: 341.0382.

*S-Methyl-S-(4-nitrophenyl)-N-p-tosyl-sulfilimine* (**3i**) [50]. Reaction of 4-nitrophenyl(methyl)sulfide **1i** (34 mg, 0.20 mmol) according to the general procedure afforded 40 mg (59%) of product **3i**, isolated as a yellow solid: mp 161–163 °C (lit. [50]; mp 150 °C); ^1^H NMR (500 MHz, CDCl_3_): δ 8.33 (d, *J* = 9.0 Hz, 2H), 7.93 (d, *J* = 9.0 Hz, 2H), 7.74 (d, *J* = 8.0 Hz, 2H), 7.21 (d, *J* = 8.0 Hz, 2H), 2.92 (s, 3H), 2.37 (s, 3H).

*S,S-Diphenyl-N-p-tosyl-sulfilimine* (**3j**) [29]. Reaction of diphenylsulfide **1j** (37 mg, 0.20 mmol) according to the general procedure afforded 40 mg (56%) of product **3j**, isolated as a white solid: mp 108–109 °C (lit. [29]; mp 109.0–109.5 °C); ^1^H NMR (500 MHz, CDCl_3_): δ 7.74 (d, *J* = 9.0 Hz, 2H), 7.64–7.59 (m, 4H), 7.52–7.41 (m, 6H), 7.14 (d, *J* = 8.5 Hz, 2H), 2.33 (s, 3H).

*S-Phenyl-S-benzyl-N-p-tosyl-sulfilimine* (**3k**) [29]. Reaction of benzyl(phenyl)sulfide **1k** (20 mg, 0.1 mmol) according to the general procedure afforded 24 mg (65%) of product **3k**, isolated as a white solid: mp 149–150 °C (lit. [29]; mp 147–148 °C); ^1^H NMR (500 MHz, CDCl_3_): δ 7.62 (d, *J* = 8.0 Hz, 2H), 7.55–7.48 (m, 3H), 7.45–7.38 (m, 2H), 7.31–7.25 (m, 1H), 7.18 (t, *J* = 7.5 Hz, 2H), 7.07 (d, *J* = 8.0 Hz, 2H), 6.98 (d, *J* = 7.5 Hz, 2H), 4.34 (d, *J* = 12.8 Hz, 1H), 4.13 (d, *J* = 12.8 Hz, 1H), 2.32 (s, 3H).

*S,S-Dibenzyl-N-p-tosyl-sulfilimine* (**3l**) [51]. Reaction of dibenzylsulfide **1l** (43 mg, 0.20 mmol) according to the general procedure afforded 51 mg (66%) of product **3l**, isolated as a white solid: mp 187–189 °C (lit. [51]; mp 191–193 °C); ^1^H NMR (500 MHz, CDCl_3_): δ 7.43 (d, *J* = 8.0 Hz, 2H), 7.36–7.26 (m, 6H), 7.22 (d, *J* = 7.5 Hz, 4H), 6.97 (d, *J* = 8.0 Hz, 2H), 4.14 (d, *J* = 13.0 Hz, 2H), 4.06 (d, *J* = 13.0 Hz, 1H), 2.32 (s, 3H).

*S,S-Dibutyl-N-p-tosyl-sulfilimine* (**3m**) [49]. Reaction of dibutylsulfide **1m** (29 mg, 0.20 mmol) according to the general procedure afforded 52 mg (79%) of product **3m**, isolated as a white solid: mp 64–65 °C (lit. [49]; mp 77.5–78.5 °C); ^1^H NMR (500 MHz, CDCl_3_): δ 7.77 (d, *J* = 7.5 Hz, 2H), 7.23 (d, *J* = 7.5 Hz, 2H), 2.88-2.78 (m, 2H), 2.77–2.68 (m, 2H), 2.38 (s, 3H), 1.59–1.46 (m, 4H), 1.36–1.22 (m, 4H), 0.82 (t, *J* = 7.0 Hz, 6H).

*S,S-Dioctyl-N-p-tosyl-sulfilimine* (**3n**). Reaction of dioctylsulfide **1n** (52 mg, 0.20 mmol) according to the general procedure afforded 77 mg (90%) of product **3n**, isolated as a white solid: mp 82 °C; IR (neat) cm^−1^: 2918, 2858, 1384, 1139, 716; ^1^H NMR (500 MHz, CDCl_3_): δ 7.79 (d, *J* = 8.3 Hz, 2H), 7.22 (d, *J* = 8.3 Hz, 2H), 2.89–2.80 (m, 2H), 2.73–2.64 (m, 2H), 2.39 (s, 3H), 1.64–1.58 (m, 4H), 1.34–1.12 (m, 20H), 0.88 (t, J = 7.3 Hz, 6H); ^13^C NMR (75 MHz, CDCl_3_): δ 141.6, 141.5, 129.1, 126.3, 49.0, 31.7, 28.9, 28.9, 28.3, 22.8, 22.6, 14.1; HRMS (ESI-TOF-positive mode): calcd for C_23_H_42_NO_2_S_2_ ([M + H])^+^: 428.2657, found: 428.2665.

*S-(p-Tosylimino)thietane* (**3o**) [52]. Reaction of thietane **1o** (15 mg, 0.20 mmol) according to the general procedure afforded 47 mg (97%) of product **3o**, isolated as a white solid: mp 103–104 °C (lit. [52]; mp 98 °C); ^1^H NMR (500 MHz, CDCl_3_): δ 7.76 (d, *J* = 8.1 Hz, 2H), 7.25 (d, *J* = 8.1 Hz, 2H), 4.26–4.08 (m, 2H), 3.70–3.57 (m, 2H), 2.62–2.16 (m, 5H).

*S-(N-p-Tosylimino)thiolane* (**3p**) [53]. Reaction of tetrahydrothiophene **1p** (18 mg, 0.20 mmol) according to the general procedure afforded 45 mg (76%) of product **3p**, isolated as a white solid: mp 131–133 °C (lit. [53]; mp 132–134 °C); ^1^H NMR (300 MHz, CDCl_3_): δ 7.79 (d, *J* = 8.1 Hz, 2H), 7.25 (d, *J* = 8.1 Hz, 2H), 3.19–3.00 (m, 4H), 2.58–2.42 (m, 2H), 2.40 (s, 3H), 2.11–1.93 (m, 2H); ^13^C NMR (75 MHz, CDCl_3_): δ 143.6, 139.1, 129.5, 126.5, 54.5, 25.6, 21.5.

*S,S-Di-tert-butyl-N-p-tosyl-sulfilimine* (**3q**) [54]. Reaction of di-*tert*-butylsulfide **1q** (29 mg, 0.20 mmol) according to the general procedure afforded 5 mg (8%) of product **3q**, isolated as a colorless oil; ^1^H NMR (500 MHz, CDCl_3_): δ 7.86 (d, *J* = 8.8 Hz, 2H), 7.33 (d, *J* = 8.8 Hz, 2H), 2.46 (s, 3H), 1.43 (s, 18H); ^13^C NMR (75 MHz, CDCl_3_): δ 144.5, 129.8, 129.5, 128.8, 49.6, 30.6, 21.7.

*S-Methyl-S-phenyl-N-p-nosyl-sulfilimine* (**3r**) [55]. Reaction of thioanisole **1a** (25 mg, 0.20 mmol) with imino-λ^3^-iodane **2b** (97 mg, 0.24 mmol) according to the general procedure afforded 61 mg (92%) of product **3r**, isolated as a white solid: mp 167 °C (lit. [55]; mp 164–165 °C); ^1^H NMR (300 MHz, CDCl_3_): δ 8.20 (d, *J* = 8.7 Hz, 2H), 8.00 (d, *J* = 8.7 Hz, 2H), 7.70 (d, *J* = 8.1 Hz, 2H), 7.62-7.46 (m, 3H), 2.93 (s, 3H); ^13^C NMR (75 MHz, CDCl_3_): δ 149.8, 149.2, 135.1, 133.1, 130.3, 127.4, 125.9, 124.0, 39.2.

*S-Methyl-S-phenyl-N-o-nosyl-sulfilimine* (**3s**). Reaction of thioanisole **1a** (25 mg, 0.20 mmol) with imino-λ^3^-iodane **2c** (97mg, 0.24 mmol) according to the general procedure afforded 33 mg (51%) of product **3s**, isolated as a colorless oil; IR (neat) cm^−1^: 3096, 3065, 3023, 2926, 1540, 1370, 1305, 1149, 1124, 852, 766; ^1^H NMR (300 MHz, CDCl_3_): δ 8.16 (d, *J* = 6.0 Hz, 1H), 7.79 (d, *J* = 8.1 Hz, 2H), 7.66–7.49 (m, 6H), 2.95 (s, 3H); ^13^C NMR (75 MHz, CDCl_3_): δ 147.5, 137.1, 136.2, 132.6, 132.2, 131.8, 130.3, 130.1, 125.8, 123.7, 39.1; HRMS (ESI-TOF-positive mode): calcd for C_13_H_13_N_2_O_4_S_2_ ([M + H])^+^: 325.0317, found: 325.0319.

*S-Methyl-S-phenyl-N-phenylsulfonyl-sulfilimine* (**3t**) [56]. Reaction of thioanisole **1a** (25 mg, 0.20 mmol) with imino-λ^3^-iodane **2d** (86 mg, 0.24 mmol) according to the general procedure afforded 38 mg (67%) of product **3t**, isolated as a white solid: mp 89–90 °C; ^1^H NMR (300 MHz, CDCl_3_): δ 7.85 (d, *J* = 7.8 Hz, 2H), 7.68 (d, *J* = 8.1 Hz, 2H), 7.57-7.32 (m, 6H), 2.85 (s, 3H).

### 4.3. Large Scale Reaction of ***1a***

Imino-λ^3^-iodane **2a** (448 mg, 1.20 mmol) was added at room temperature to a stirred mixture of thioanisole **1a** (124 mg, 1.00 mmol) and I_2_ (5 mg, 0.02 mmol) in dichloromethane (10.0 mL). The reaction was stirred at room temperature for 24 h. After reaction, 5% aqueous Na_2_S_2_O_3_ (25 mL) was added to the mixture and the solution was extracted with ethyl acetate. The organic layer was dried over anhydrous Na_2_SO_4_ and concentrated under reduced pressure. The residue was separated by column chromatography using the Hexane-EtOAc (1:1) to afford the pure product **3a** in 76% (223 mg).

### 4.4. Reaction of Triphenylphosphine 4

Imino-λ^3^-iodane **2a** (90 mg, 0.24 mmol) was added at room temperature to a stirred mixture of triphenylphosphine **4** (52 mg, 0.20 mmol) and I_2_ (1 mg, 0.004 mmol) in dichloromethane (2.0 mL). The reaction was stirred at room temperature for 24 h. After reaction, 5% aqueous Na_2_S_2_O_3_ (2.5 mL) was added to the mixture and the solution was extracted with ethyl acetate. The organic layer was dried over anhydrous Na_2_SO_4_ and concentrated under reduced pressure. The residue was separated by preparative TLC using the Hexane-EtOAc (1:1) to afford the pure product **5** in 98% (84 mg).

*N-(p-Tosyl)iminotriphenylphosphorane* (**5**) [39]. Isolated as a white solid: mp 190 °C (lit. [39]; mp 185.5–186.2 °C); ^1^H NMR (500 MHz, CDCl_3_): δ 7.44 (dd, *J* = 12.3 Hz, 7.8 Hz, 2H), 7.57 (t, *J* = 7.8 Hz, 3H), 7.50 (d, *J* = 7.8 Hz, 2H), 7.46 (dt, *J* = 7.8 Hz, 3.0 Hz, 6H), 7.00 (d, *J* = 7.8 Hz, 2H), 2.23 (s, 3H).

Single crystals of product **5** suitable for X-ray crystallographic analysis were obtained by slow crystallization from dichloromethane solution. X-ray diffraction data for **5** was identical to the previously reported compound [37].

## 5. Conclusions

In conclusion, we have developed the metal-free methodology of the sulfonyl group transfer reaction of sulfides **1** or triphenylphosphine **4** using imino-λ^3^-iodanes **2** in the presence of a catalytic amount of I_2_. The novel procedure gives the corresponding (*N-*sulfonyl)-sulfilimines **3** or (*N*-tosylimino)-triphenylphosphorane **5** in moderate to good yields. According to the blank experiment study, the reaction mechanism most likely involved the amidyl radical species, which are generated from imino-λ^3^-iodanes **2** and I_2_.

## Figures and Tables

**Table 1:**
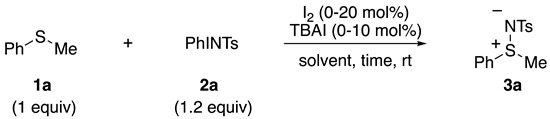
Optimization of imination reaction of thioanisole **1a** using (*N*-tosylimino)-phenyl-λ^3^-iodane **2a** ^1^.

Entry	Time (h)	Solvent	I_2_ (mol%)	TBAI (mol%)	3a Yield (%) ^2^
1	3	MeCN	20	10	81 (80)
2	3	Hexane	20	10	15
3	3	AcOEt	20	10	49
4	3	MeOH	20	10	77
5	3	THF	20	10	4
6	3	Et_2_O	20	10	Trace
7	3	PhH	20	10	47
8	3	CCl_4_	20	10	7
9	3	CH_2_Cl_2_	20	10	83 (83)
10	3	CH_2_Cl_2_	20	none	84
11	3	CH_2_Cl_2_	none	10	7
12	3	CH_2_Cl_2_	none	none	5
13	3	CH_2_Cl_2_	10	none	72
14	24	CH_2_Cl_2_	10	none	87
15	24	CH_2_Cl_2_	5	none	91
16	24	CH_2_Cl_2_	2	none	92 (88)
17	24	CH_2_Cl_2_	1	none	68
18	48	CH_2_Cl_2_	1	none	66
19 ^3^	24	CH_2_Cl_2_	2	none	77

^1^ Reaction conditions: thioanisole **1a** (0.20 mmol, 1 equiv.), iminoiodane **2a** (0.24 mmol, 1.2 equiv.), and I_2_ (0–20 mol%) with TBAI (0–10 mol%) stirred in a solvent (2 mL) at room temperature for 3–48 h. ^2^ Yields of product **3a** determined from ^1^H NMR spectra of reaction mixtures are shown (numbers in parentheses show isolated yield of **3a**). ^3^ The reaction was performed under dark conditions.

**Table 2:**
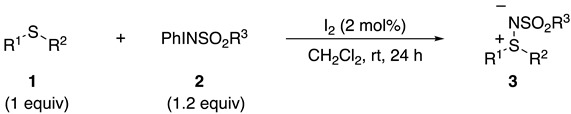
Metal-free imination reaction of thioanisoles **1** with imino-λ^3^-iodane **2** in the presence of I_2_^1^.

Entry	1 R^1^, R^2^	2 R^3^	3 Yield(%) ^2^
1	**1a** Ph, Me	**2a***p*-Tol	**3a** 88% (76%) ^3^
2	**1b***p*-tol, Me	**2a***p*-Tol	**3b** 72%
3	**1c***p*-MeOC_6_H_4_, Me	**2a***p*-Tol	**3c** 78%
4	**1d***p*-ClC_6_H_4_, Me	**2a***p*-Tol	**3d** 79%
5	**1e***m*-ClC_6_H_4_, Me	**2a***p*-Tol	**3e** 80%
6	**1f***o*-ClC_6_H_4_, Me	**2a***p*-Tol	**3f** 68%
7	**1g***p*-BrC_6_H_4_, Me	**2a***p*-Tol	**3g** 70%
8	**1h***p*-NCC_6_H_4_, Me	**2a***p*-Tol	**3h** 86%
9	**1i***p*-NO_2_C_6_H_4_, Me	**2a***p*-Tol	**3i** 59%
10	**1j** Ph, Ph	**2a***p*-Tol	**3j** 56%
11	**1k** Ph, Bn	**2a***p*-Tol	**3k** 65%
12	**1l** Bn, Bn	**2a***p*-Tol	**3l** 66%
13	**1m***n*Bu, *n*Bu	**2a***p*-Tol	**3m** 79%
14	**1n***n*Octyl, *n*Octyl	**2a***p*-Tol	**3n** 90%
15	**1o** –(CH_2_)_3_–	**2a***p*-Tol	**3o** 97%
16	**1p** –(CH_2_)_4_–	**2a***p*-Tol	**3p** 76%
17	**1q***t*Bu, *t*Bu	**2a***p*-Tol	**3q** 8%
18	**1a** Ph, Me	**2b***p*-NO_2_C_6_H_4_	**3r** 92%
19	**1a** Ph, Me	**2c***o*-NO_2_C_6_H_4_	**3s** 51%
20	**1a** Ph, Me	**2d** Ph	**3t** 67%

^1^ All reactions were performed using sulfide **1** (0.20 mmol, 1 equiv.) and imino-λ^3^-iodane **2** (0.24 mmol, 1.2 equiv.) using I_2_ (2 mol%) in dichloromethane (2.0 mL) at room temperature for 24 h. ^2^ Yields of isolated products. ^3^ Large scale experiment: thioanisole **1a** (1.0 mmol, 1 equiv.) and imino-λ^3^-iodane **2** (1.2 mmol, 1.2 equiv.) using I_2_ (2 mol%) in the dichloromethane (10 mL) were stirred for 24 h at room temperature.

## Supplementary Materials

The following are available online. The ^1^H and ^13^C NMR spectra of **3a–t**, **5**, and X-ray structure data of **5** can be found in the SI.
