# Supplementary material for: Sulfonylimino Group Transfer Reaction Using Imino-λ3-iodanes with I2 as Catalyst Under Metal-free Conditions
Source: Molecules. 2019 Mar 11;24(5):979. doi: 10.3390/molecules24050979 (PMC6429398; doi:10.3390/molecules24050979)

Supporting Information

Sulfonylimino group transfer reaction using imino-λ³-iodanes with I_2 under metal-free conditions

Akira Yoshimura [1,2,*], Cody L. Makitalo [2], Melissa E. Jarvi [2], Michael T. Shea [2], Pavel S. Postnikov [1], Gregory T. Rohde [3], Viktor V. Zhdankin [2], Akio Saito [4], and Mekhman S. Yusubov [1]

[1] The Tomsk Polytechnic University, 634050 Tomsk, Russia
[2] Department of Chemistry and Biochemistry, University of Minnesota Duluth, MN 55812, USA
[3] Marshall School, Duluth, Minnesota 55811 USA
[4] Division of Applied Chemistry, Institute of Engineering, Tokyo University of Agriculture and Technology, 2-24-16 Naka-cho, Koganei, Tokyo 184-8588, Japan

* Correspondence: yoshimura@tpu.ru; ayoshimu@d.umn.edu

Table of Contents: **pp**

1. ***X-Ray crystallography data of 5*** **p.2**

2. ***Spectra of products*** **p.3**

X-Ray crystallography data of 5

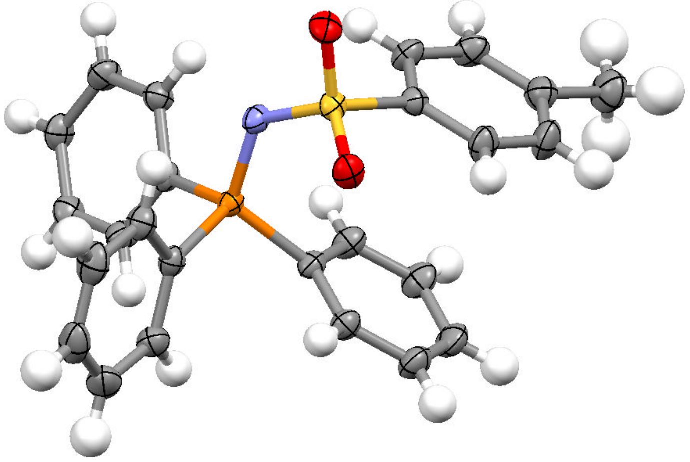

Figure S1. X-Ray crystal structure of 5.

Single crystals of product **5** suitable for X-ray crystallographic analysis were obtained by slow crystallization from dichloromethane solution. X-ray diffraction data for **5** were collected on Rigaku RAPID II Image Plate using graphite-monochromated MoKα radiation (λ = 0.71073 Å) at 173 K. The unit cell of 8.9064(5), 10.5791(6), 13.0086(9), 99.870(7), 107.920(8) and bond metrics for 5 matched the previously reported compound.[1]

Reference

Cameron, A. F.; Hair, N. J.; Morris, D. G. Structural investigations of ylides. IV. Crystal and molecular structure of N-(*p*-toluenesulfonyl)iminotriphenylphosphorane. Acta Crystallogr., Sect. B **1974**, *30*, 221-225.

^{1}H NMR (500 MHz, $CDCl_3$)

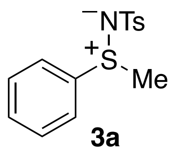

3a

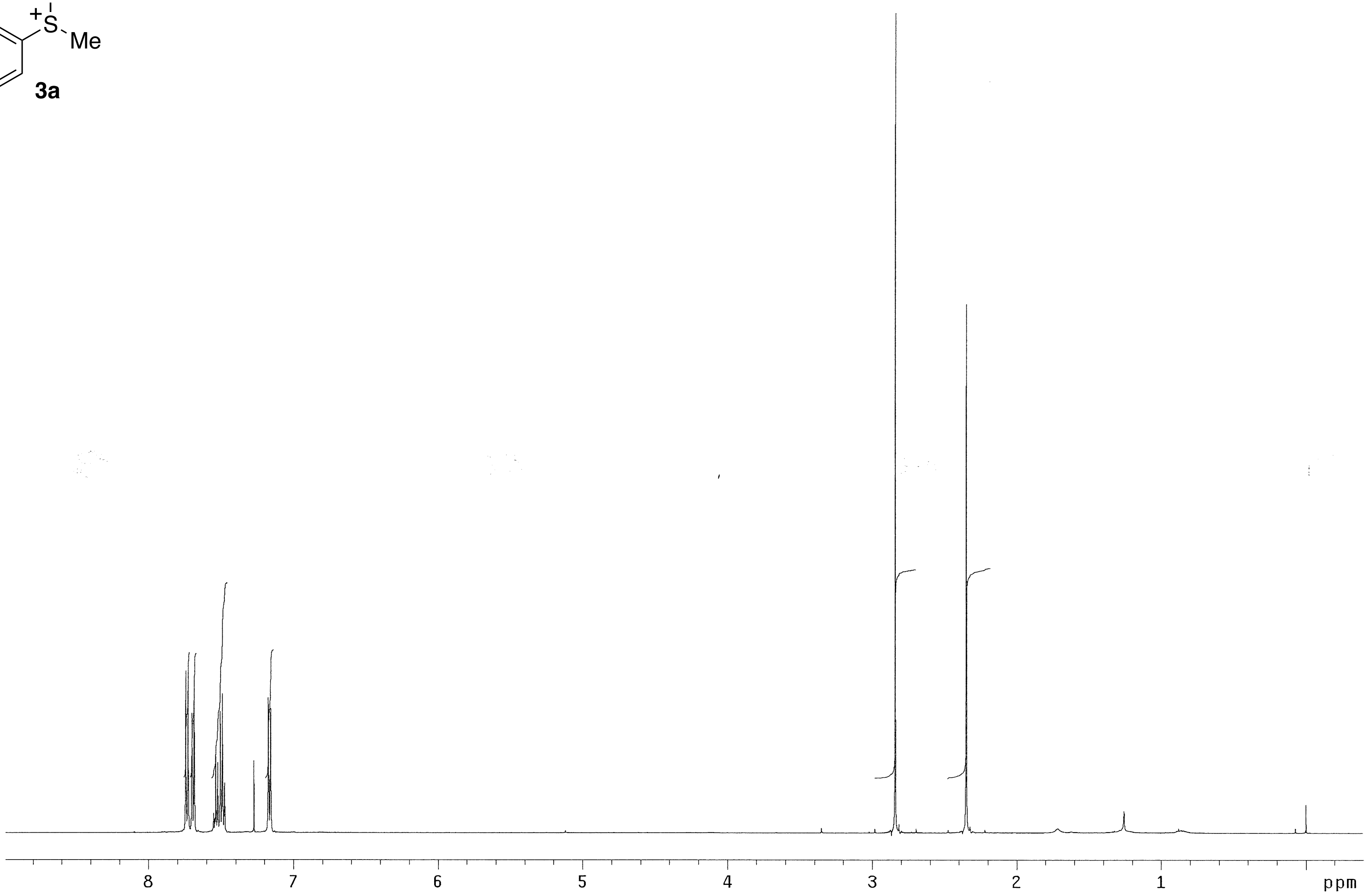

^{1}H NMR (500 MHz, $CDCl_3$)

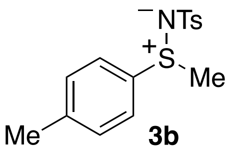

3b

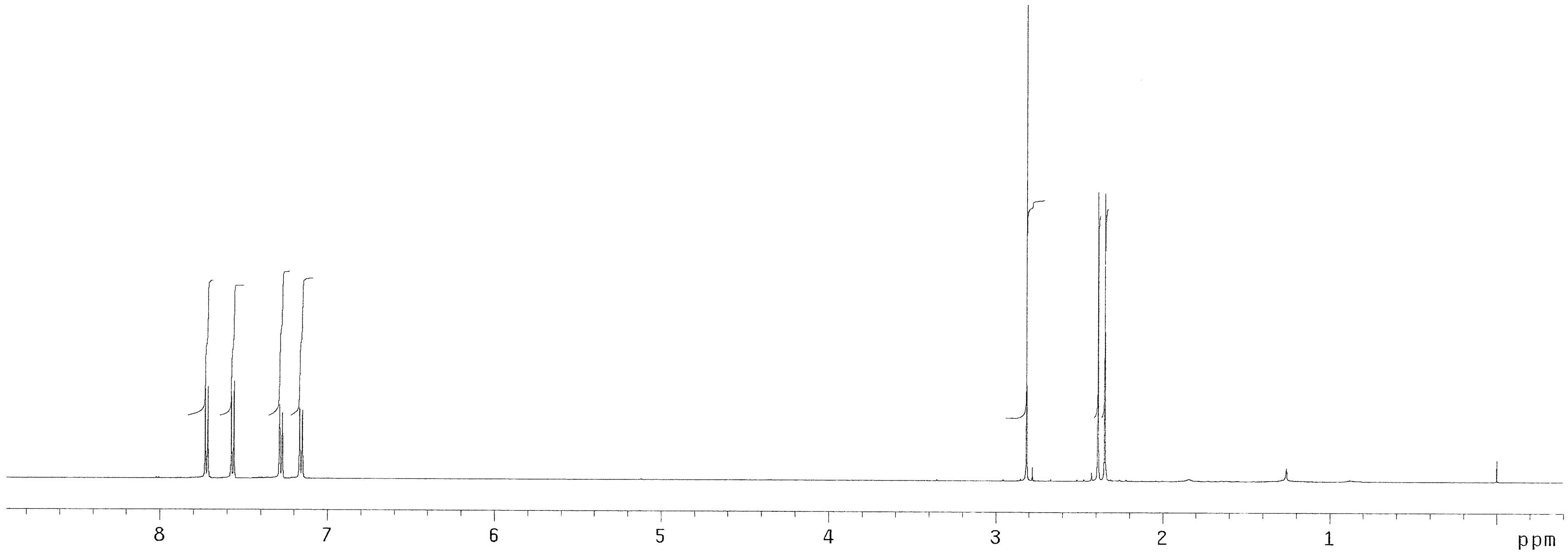

^{1}H NMR (500 MHz, $CDCl_3$)

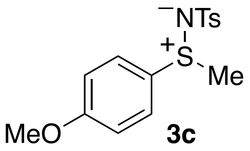

3c

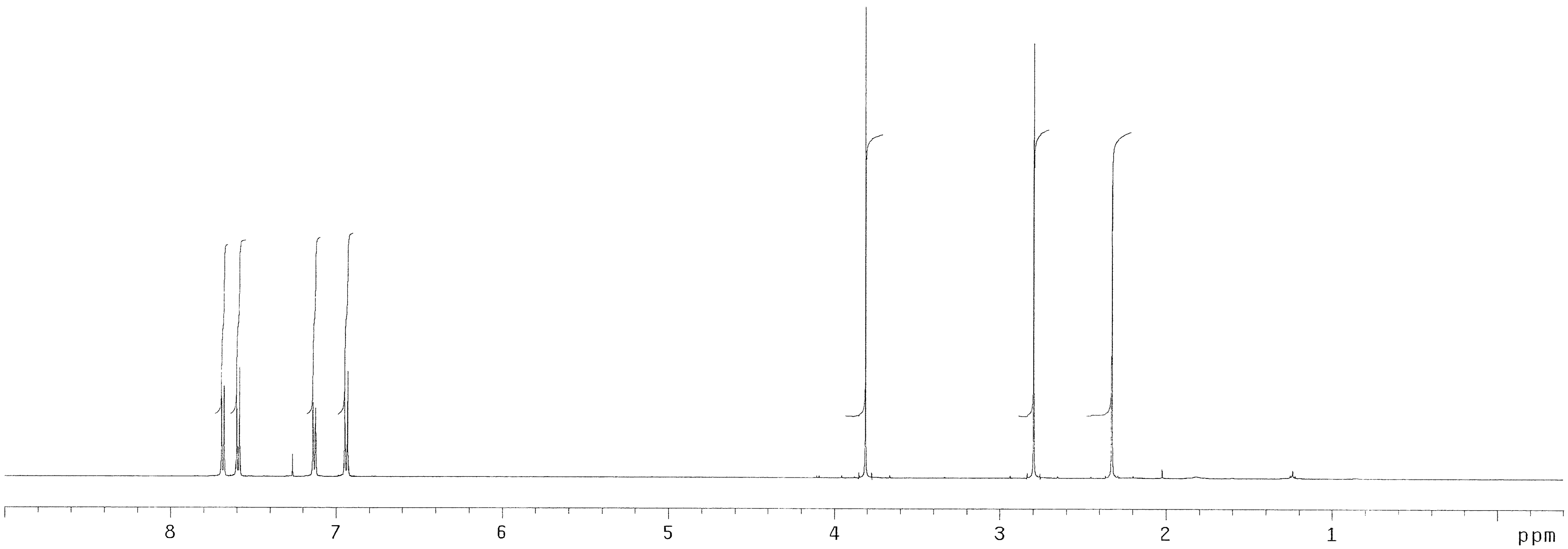

^{1}H NMR (500 MHz, $CDCl_3$)

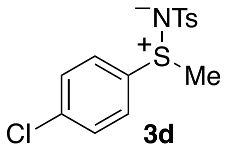

3d

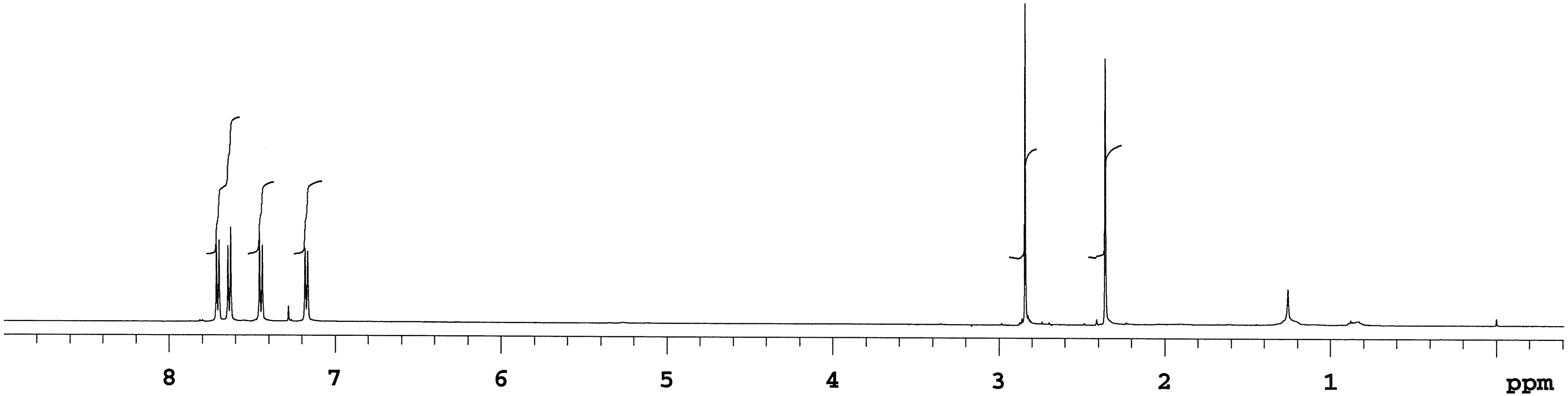

^{1}H NMR (500 MHz, $CDCl_3$)

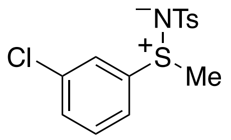

3e

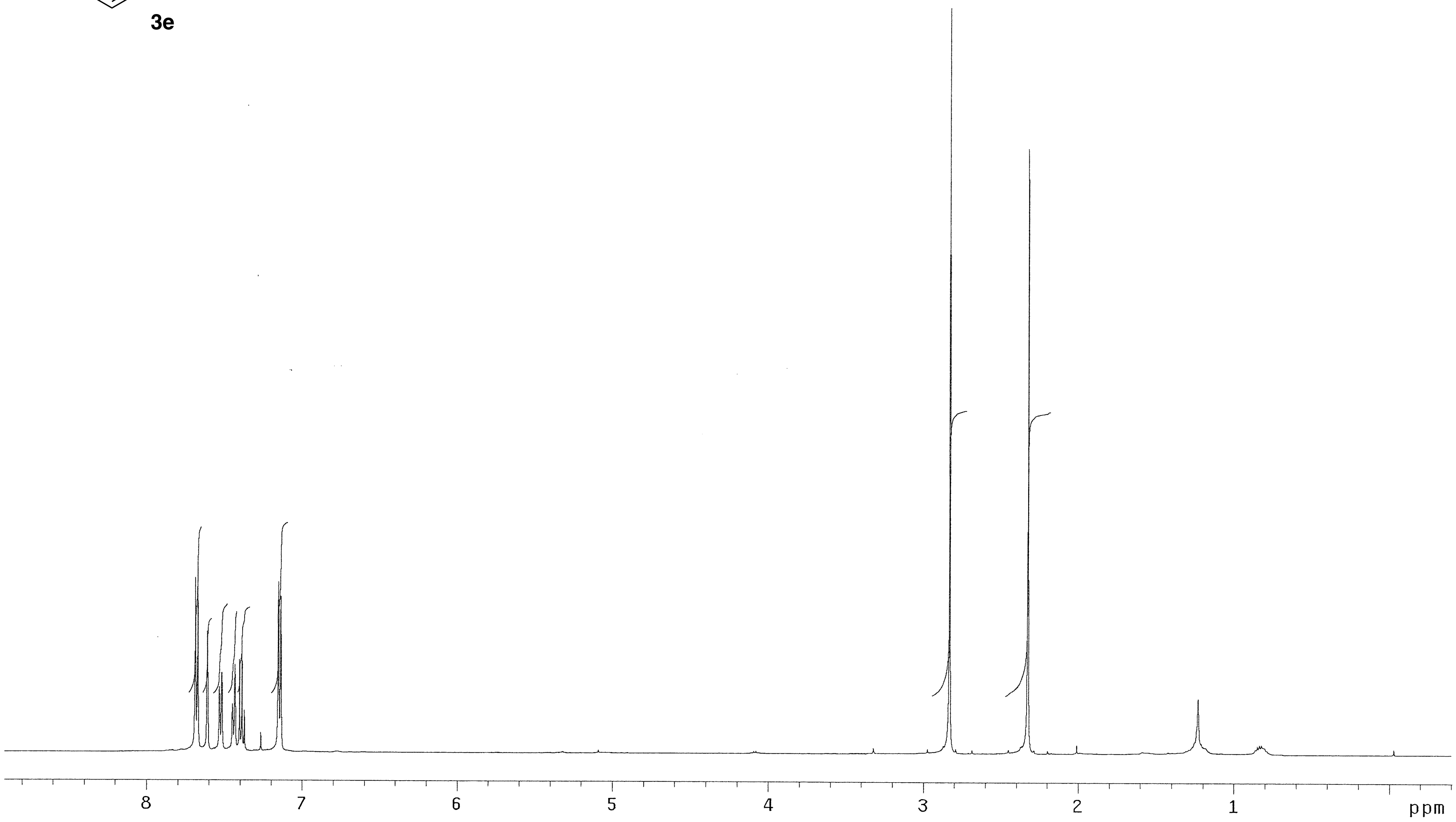

^{1}H NMR (500 MHz, $CDCl_3$)

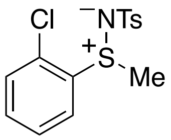

3f

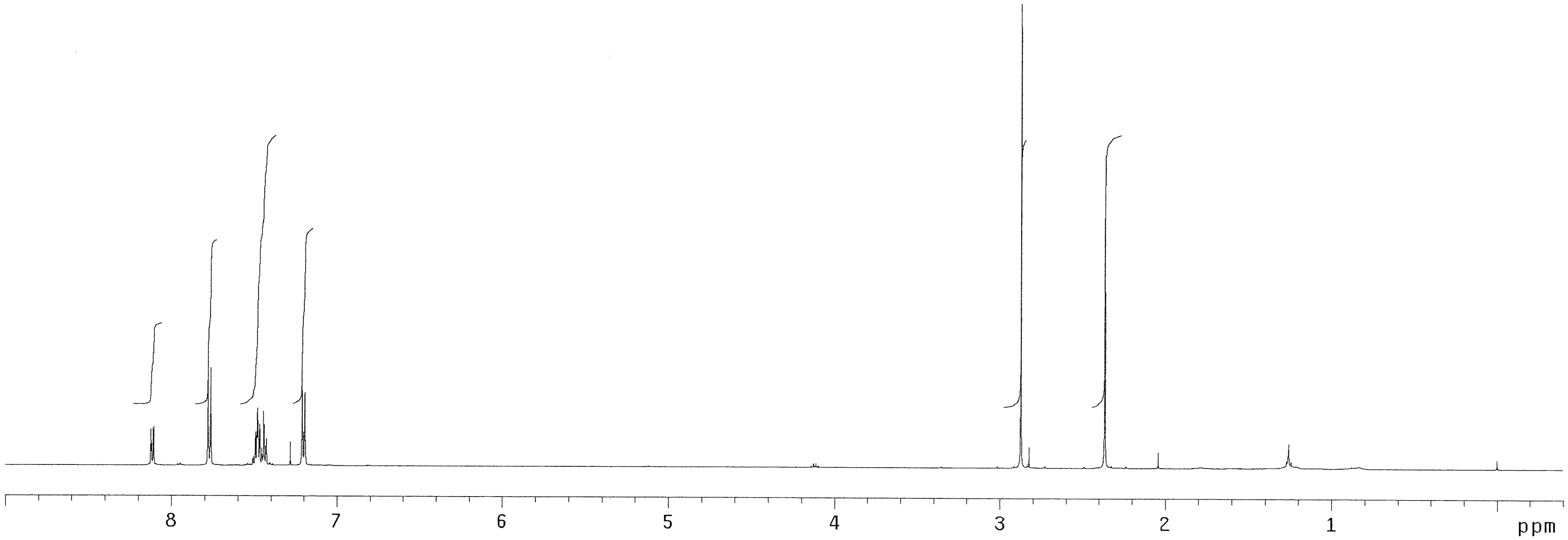

^{1}H NMR (500 MHz, $CDCl_3$)

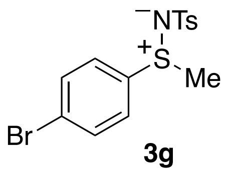

3g

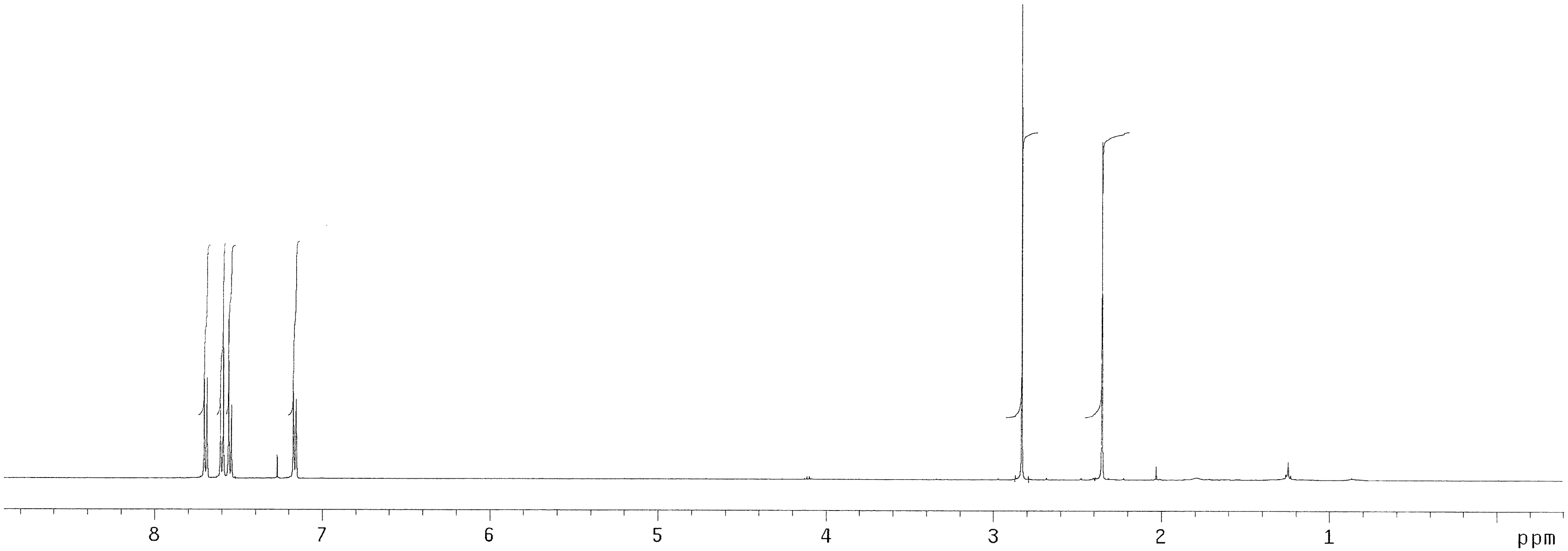

^{1}H NMR (500 MHz, $CDCl_3$)

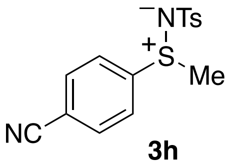

3h

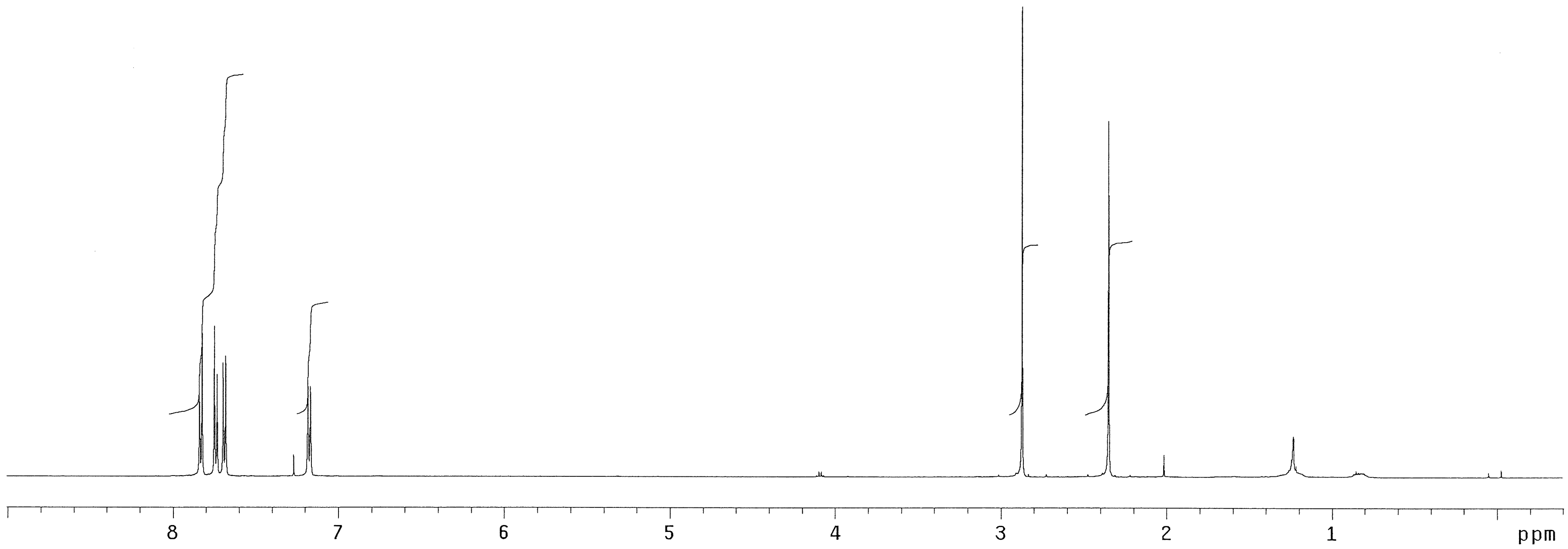

^{13}C NMR (75 MHz, $CDCl_3$)

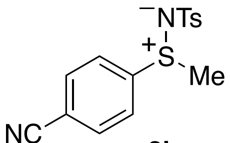

3h

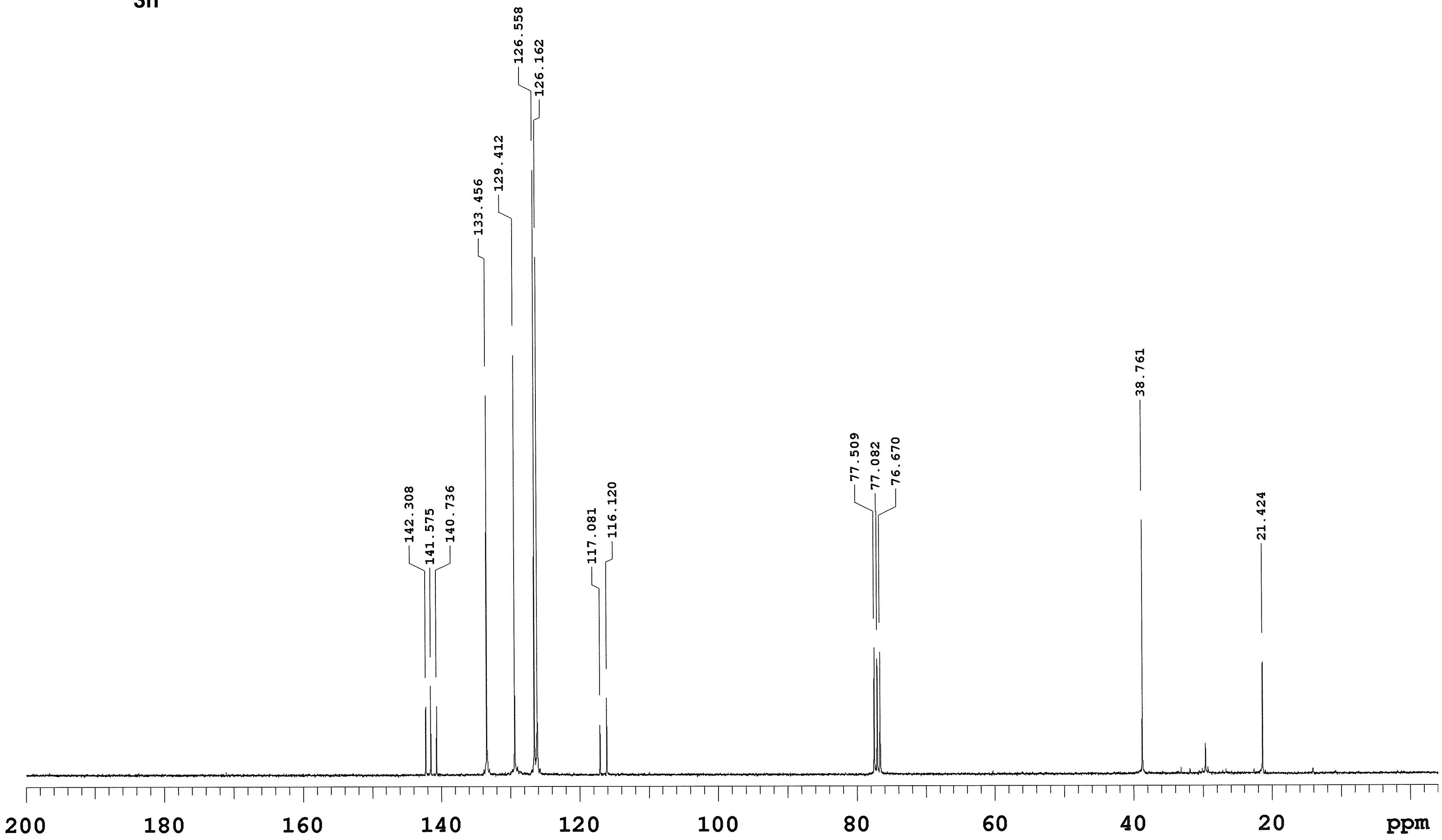

^{1}H NMR (500 MHz, $CDCl_3$)

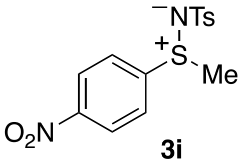

3i

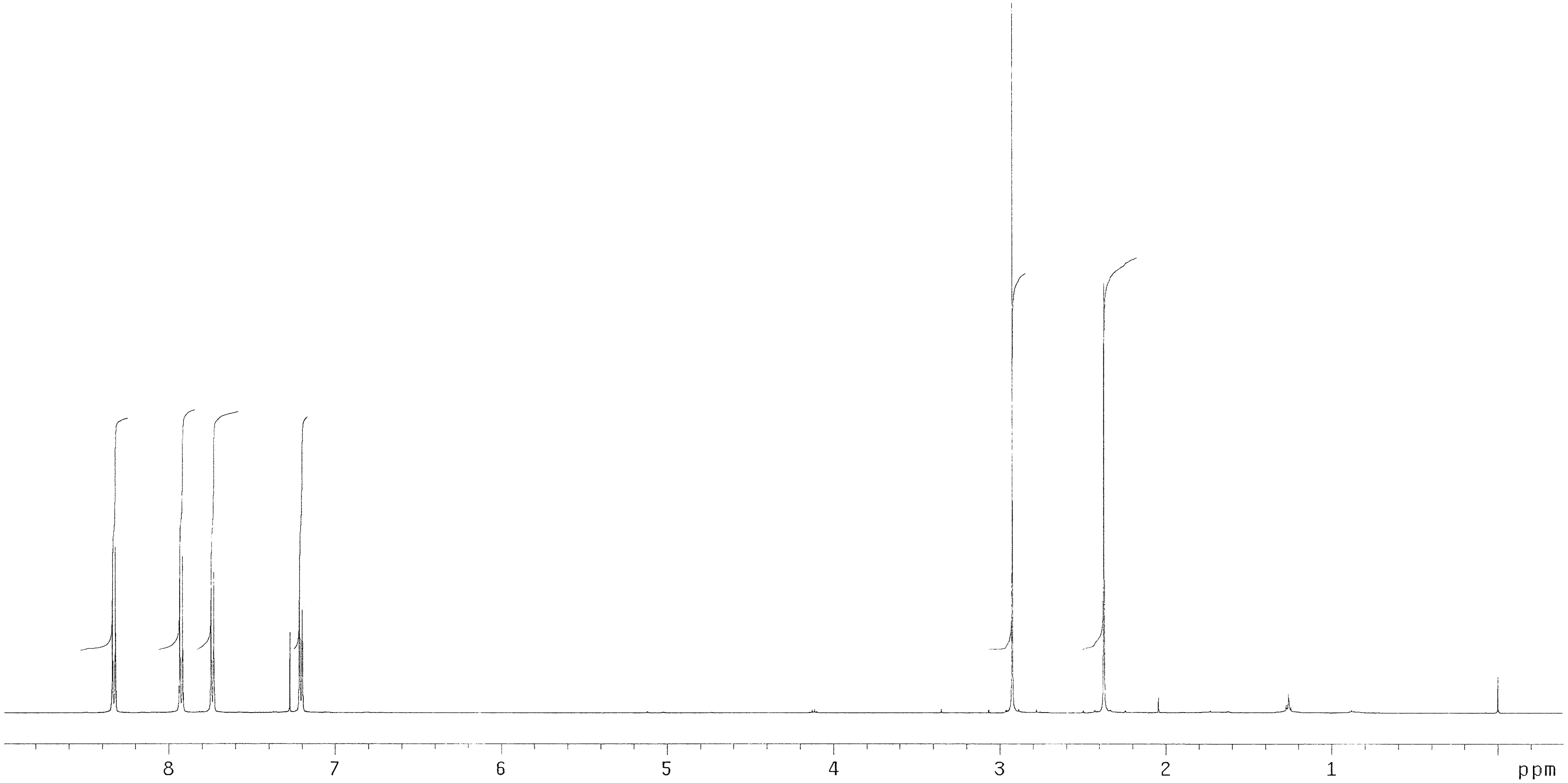

^{1}H NMR (500 MHz, $CDCl_3$)

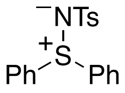

3j

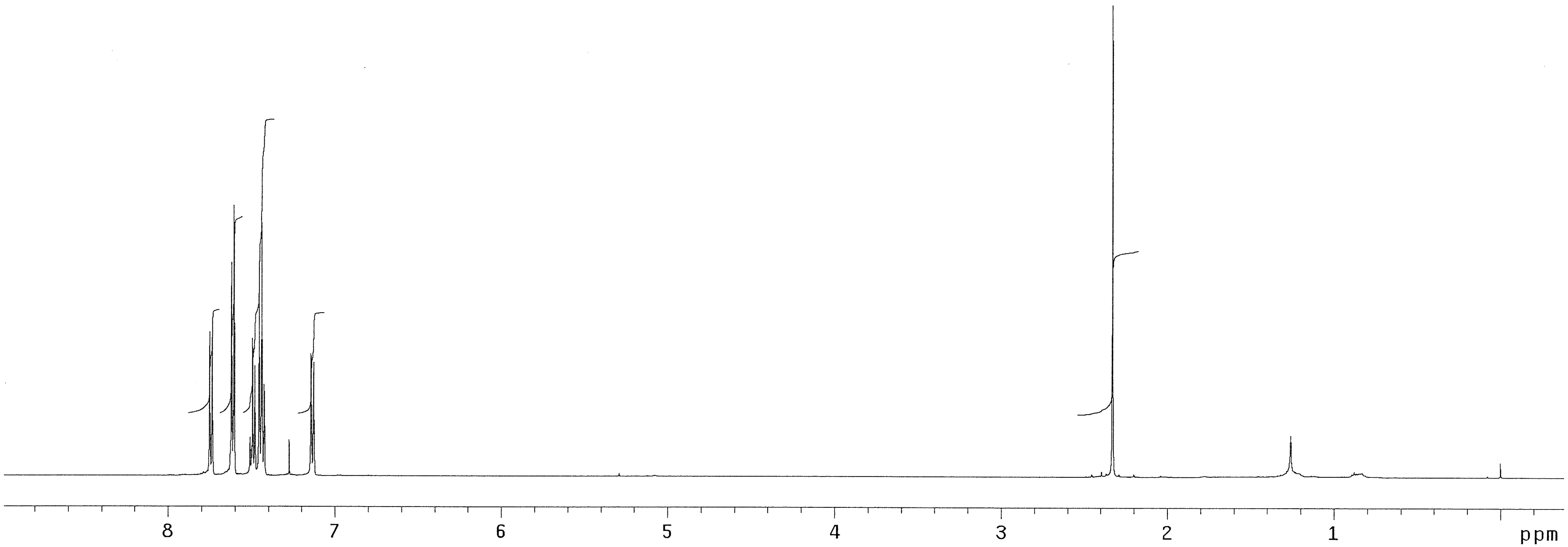

^{1}H NMR (500 MHz, $CDCl_3$)

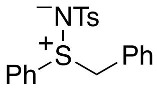

3k

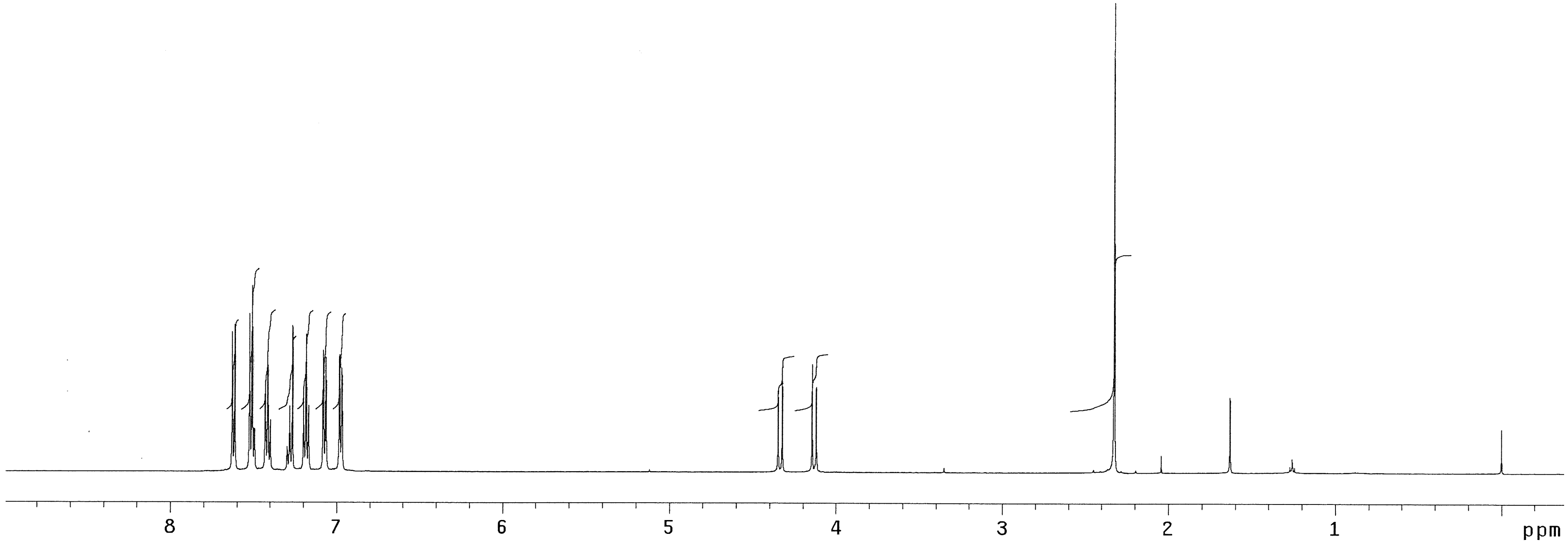

^{1}H NMR (500 MHz, $CDCl_3$)

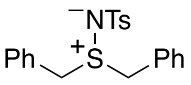

3l

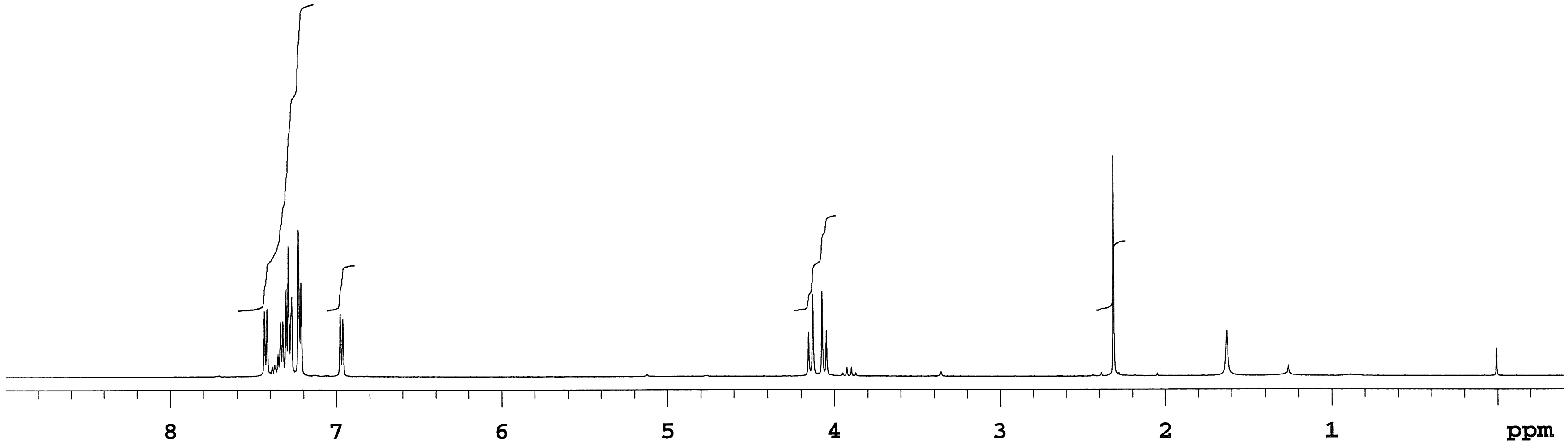

^{1}H NMR (500 MHz, $CDCl_3$)

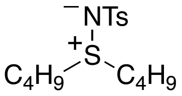

3m

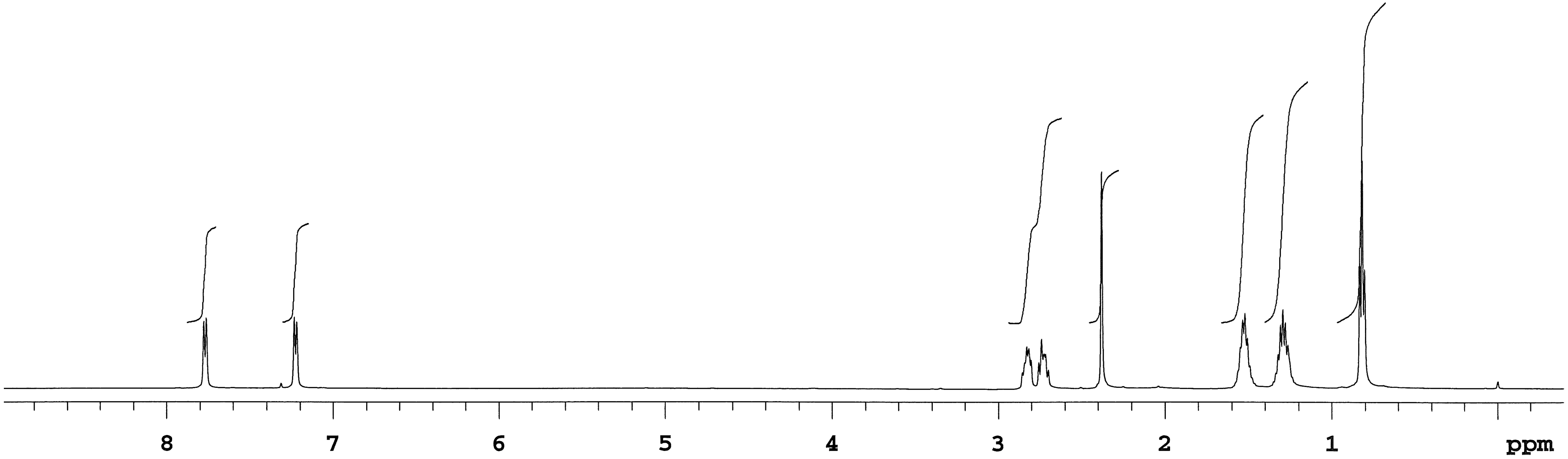

^{1}H NMR (500 MHz, $CDCl_3$)

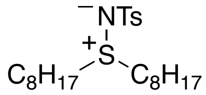

3n

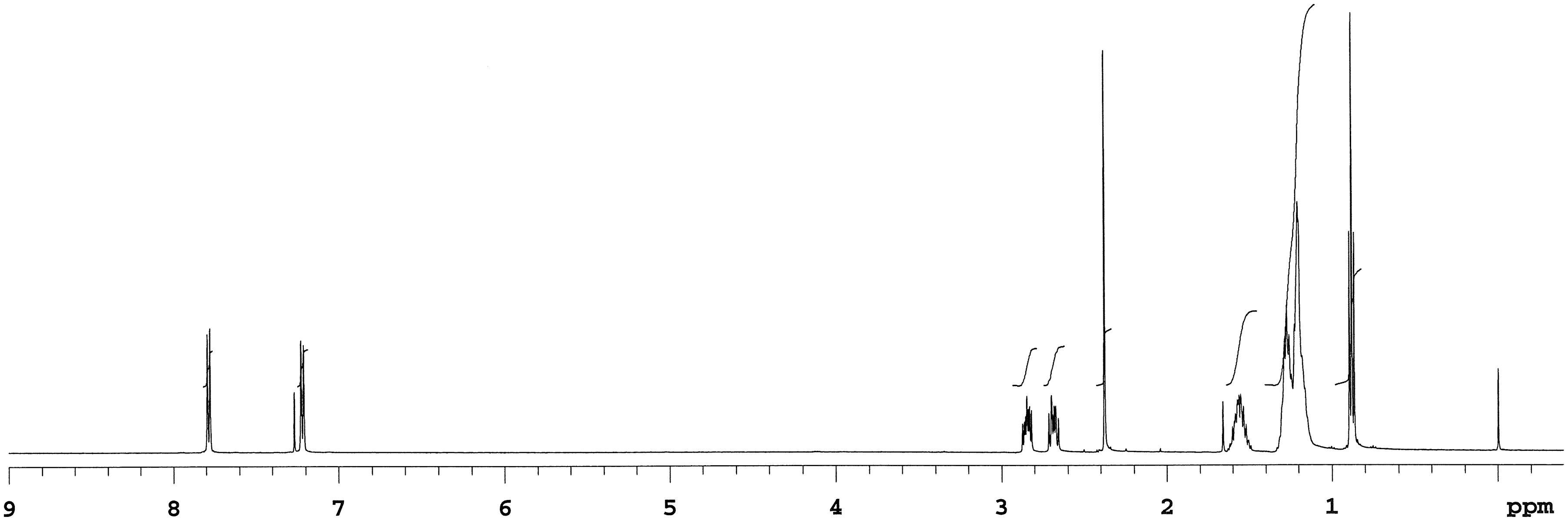

^{13}C NMR (75 MHz, $CDCl_3$)

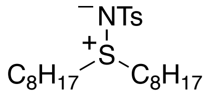

3n

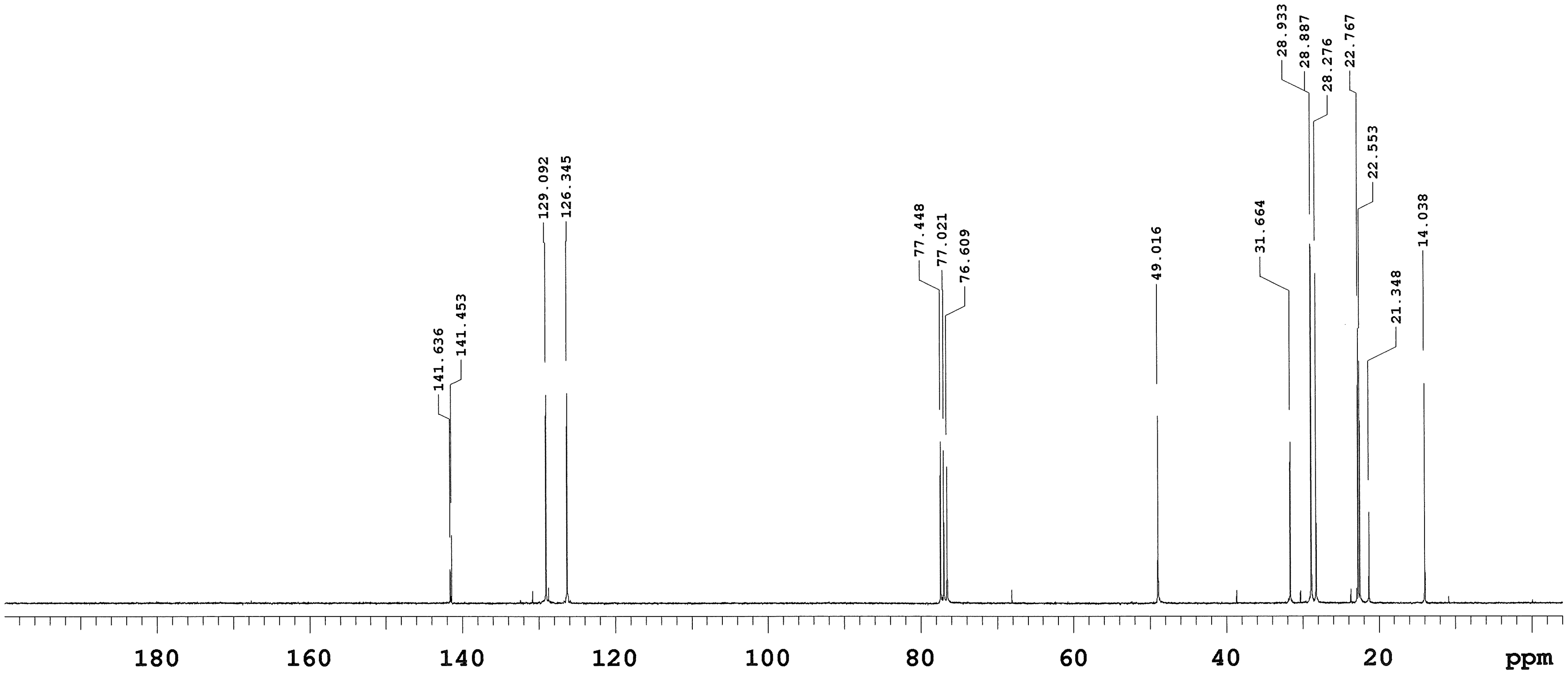

^{1}H NMR (300 MHz, $CDCl_3$)

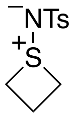

3o

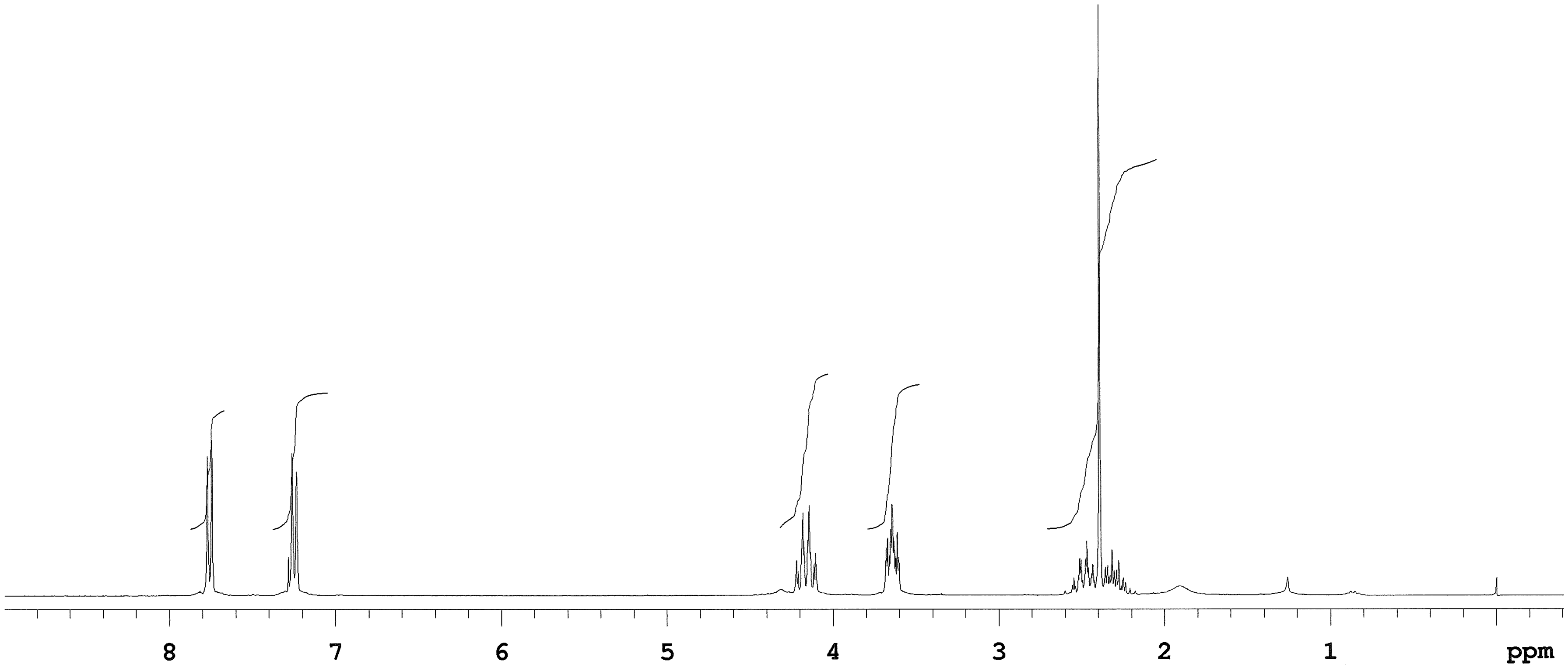

^{1}H NMR (300 MHz, $CDCl_3$)

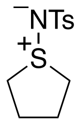

3p

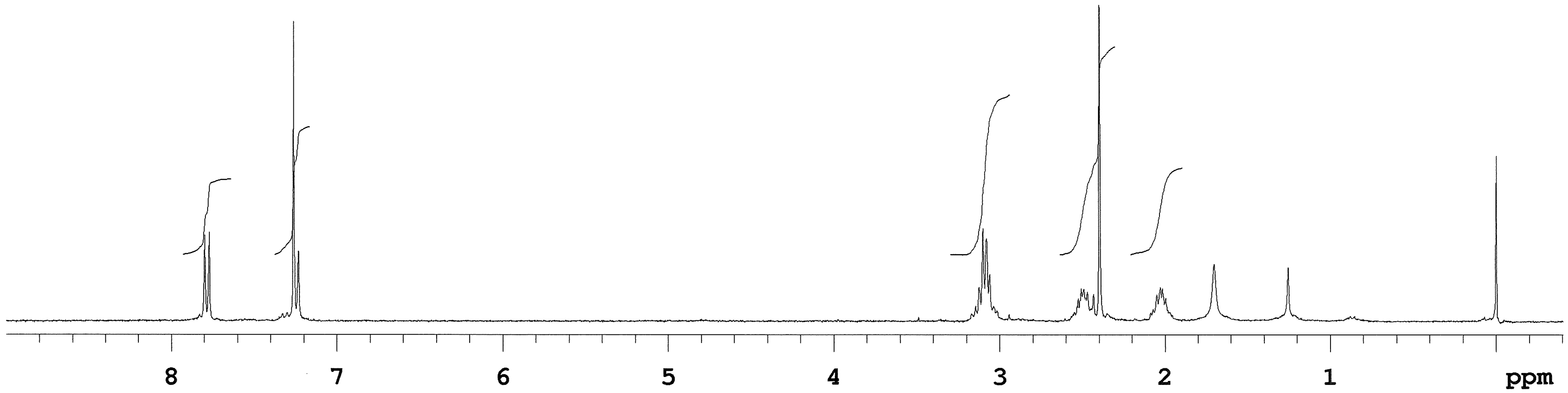

^{13}C NMR (75 MHz, $CDCl_3$)

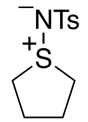
3p

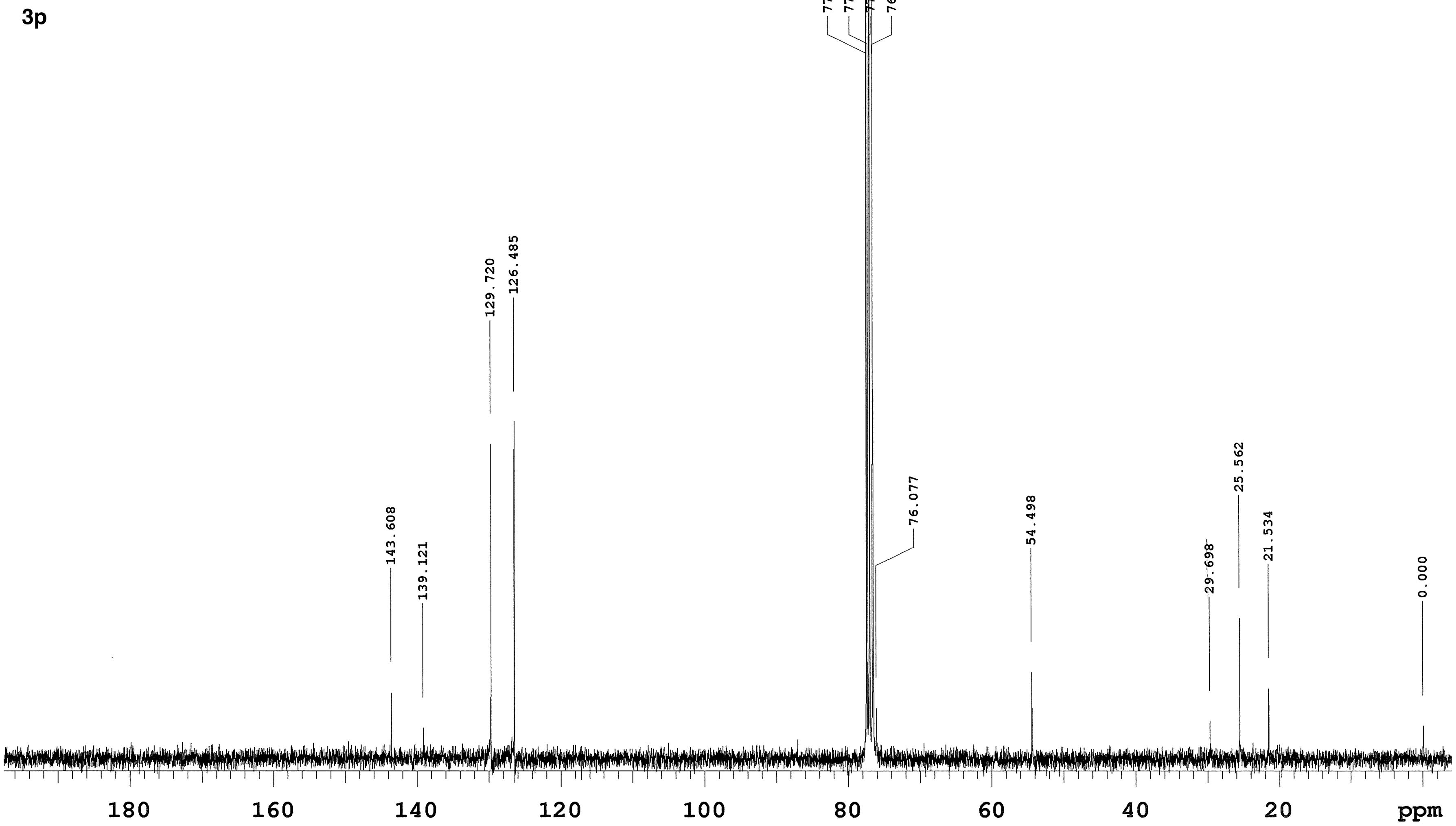

^{1}H NMR (500 MHz, $CDCl_3$)

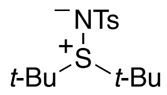

3q

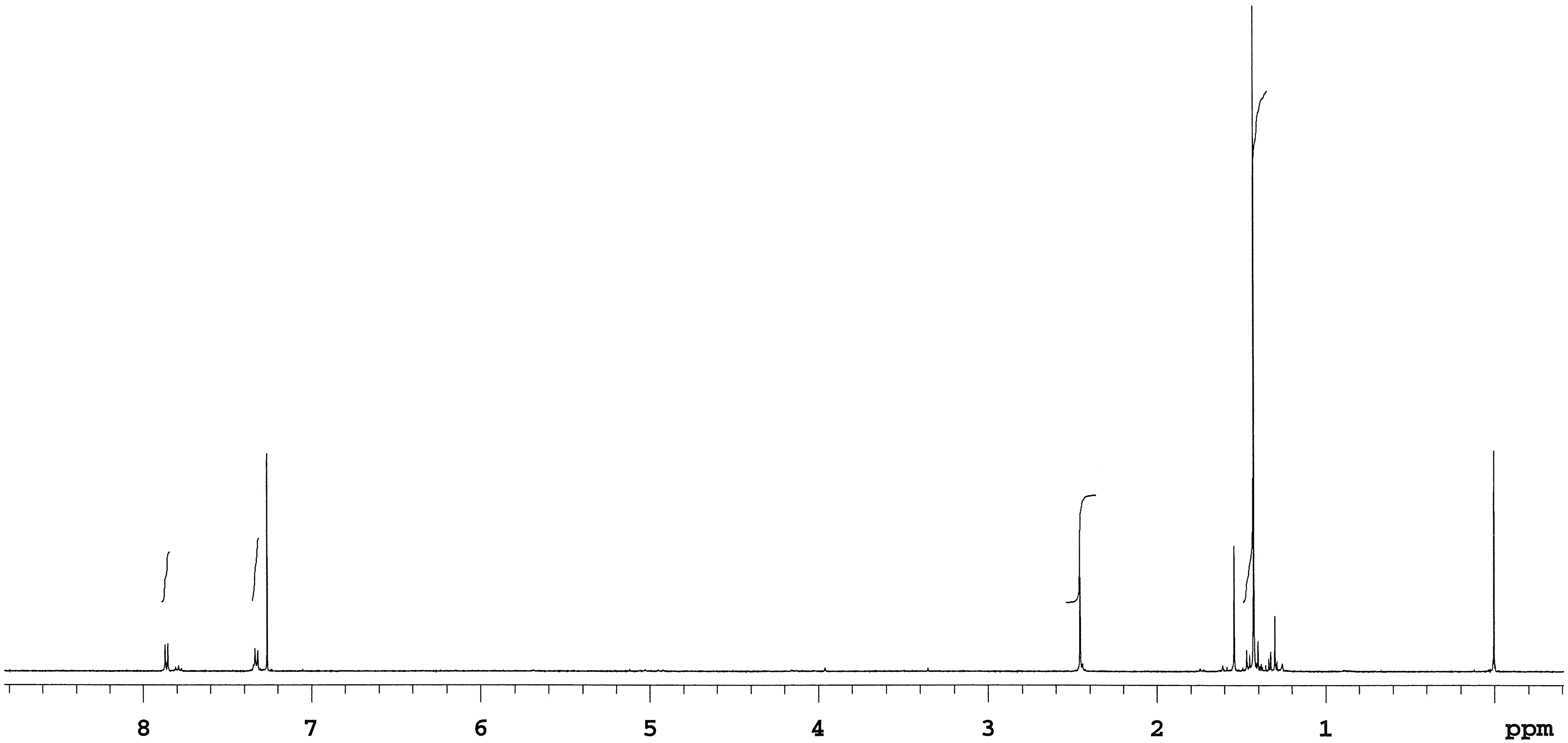

^{13}C NMR (75 MHz, $CDCl_3$)

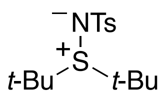

3q

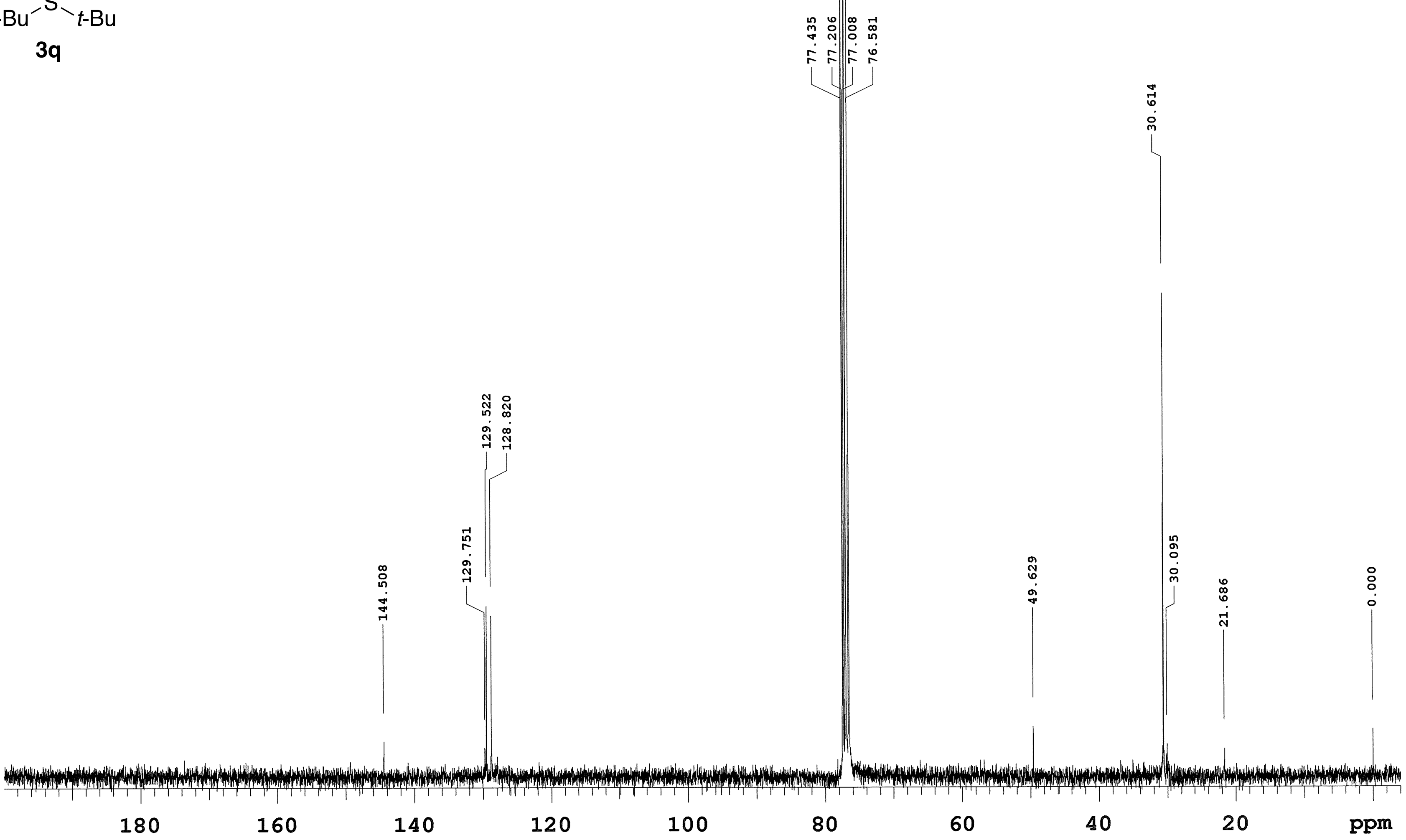

^{1}H NMR (300 MHz, $CDCl_3$)

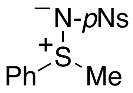

3r

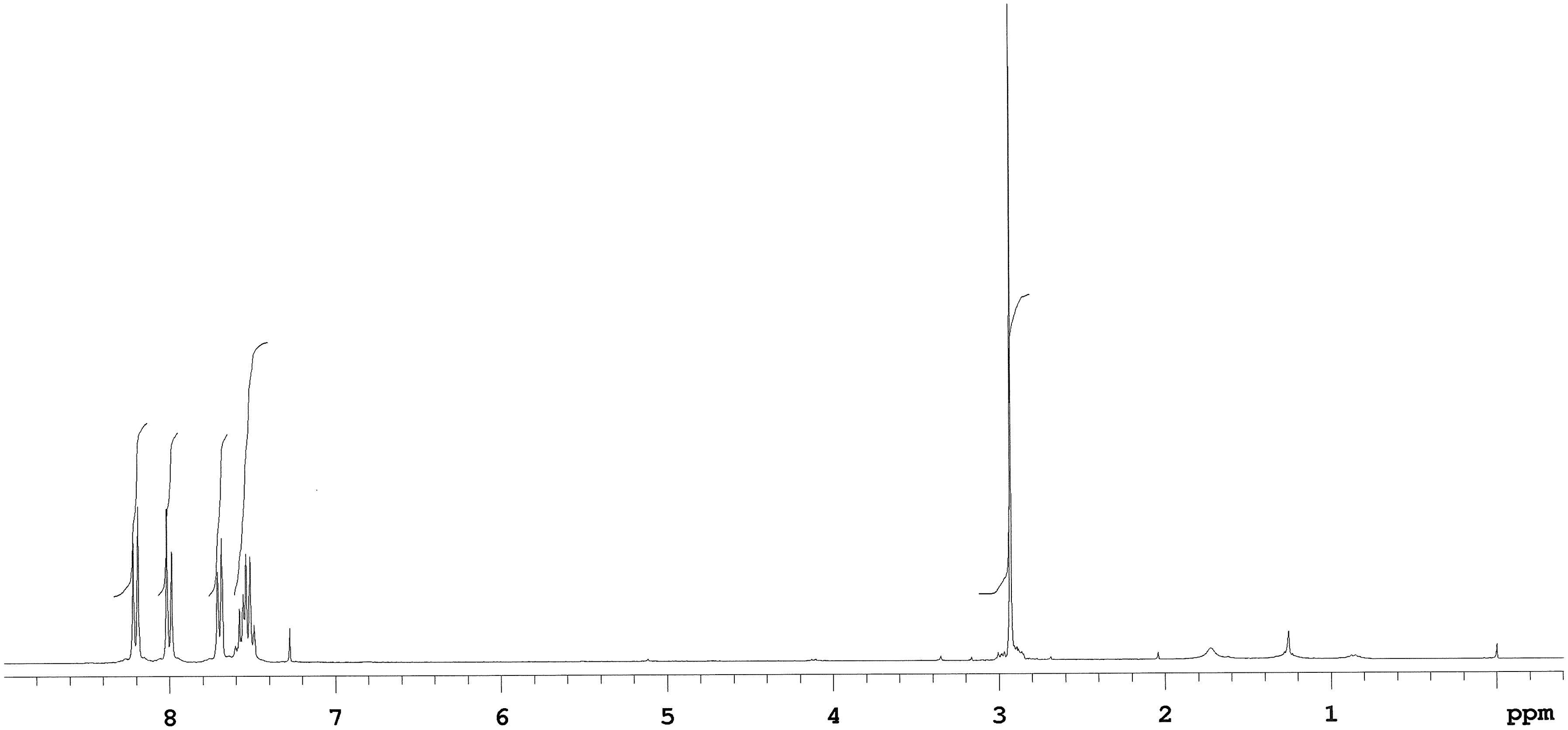

^{13}C NMR (75 MHz, $CDCl_3$)

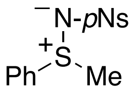

3r

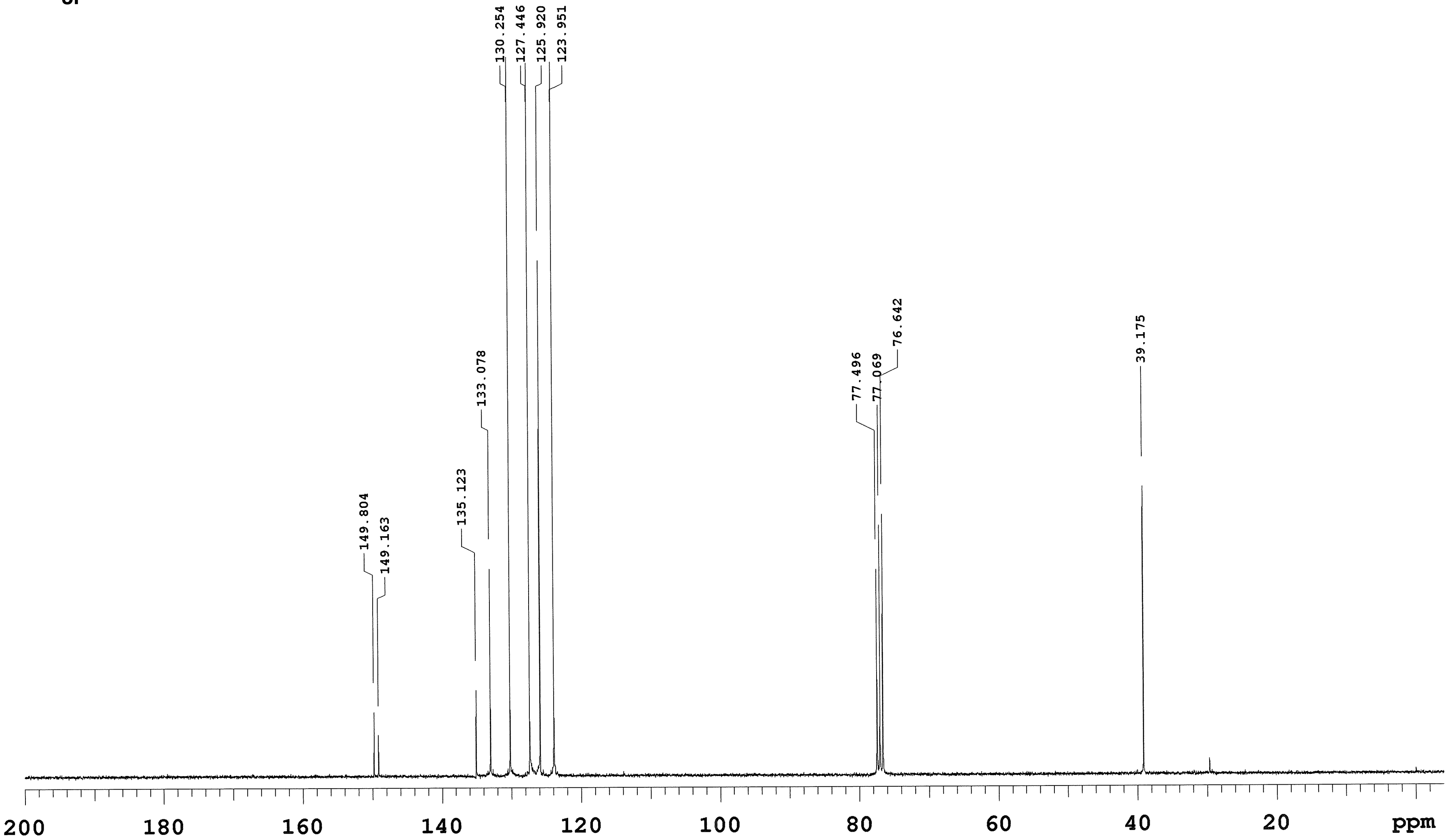

^{1}H NMR (300 MHz, $CDCl_3$)

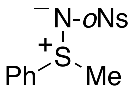

3s

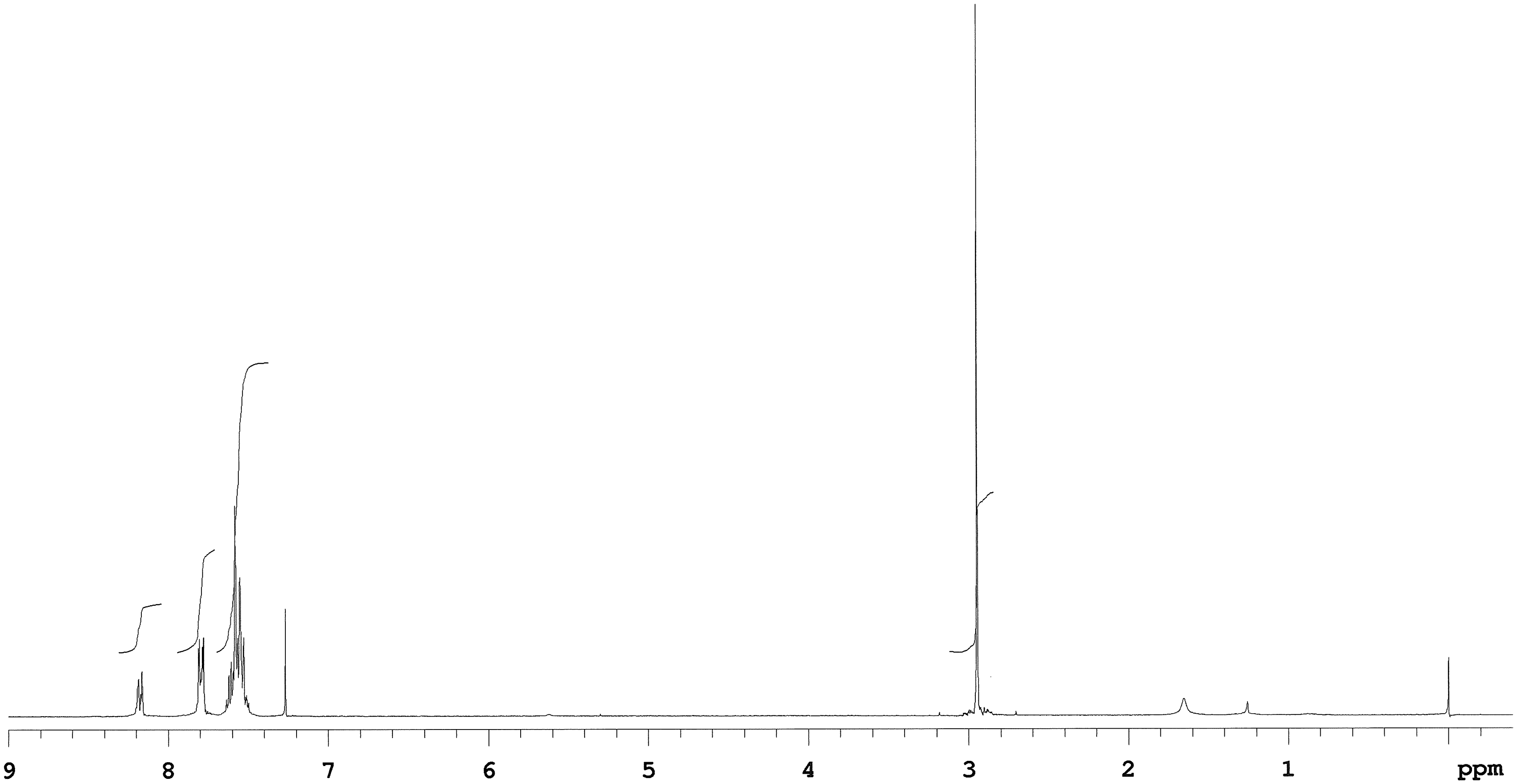

^{13}C NMR (75 MHz, $CDCl_3$)

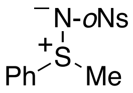

3s

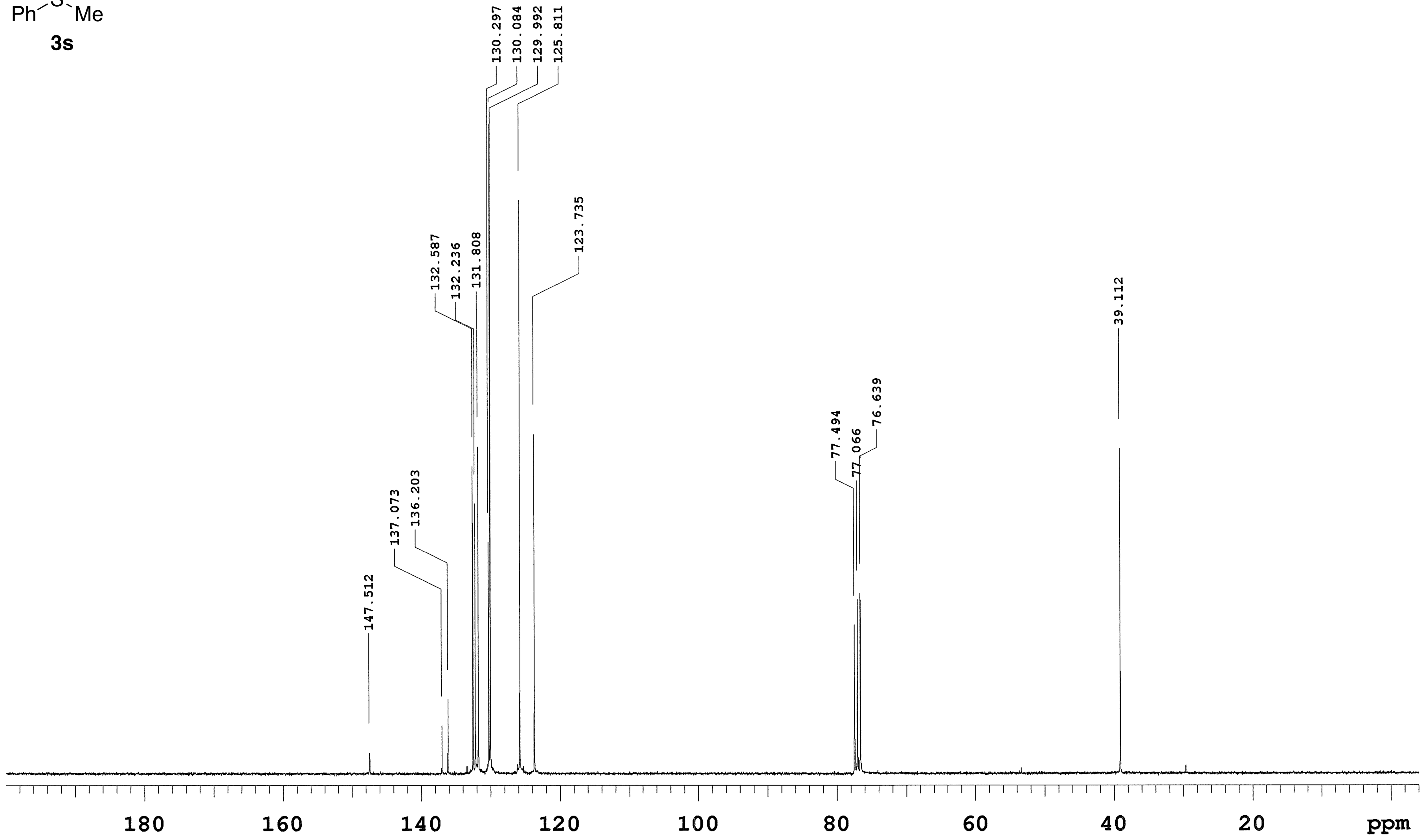

^{1}H NMR (300 MHz, $CDCl_3$)

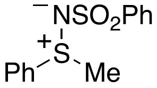

3t

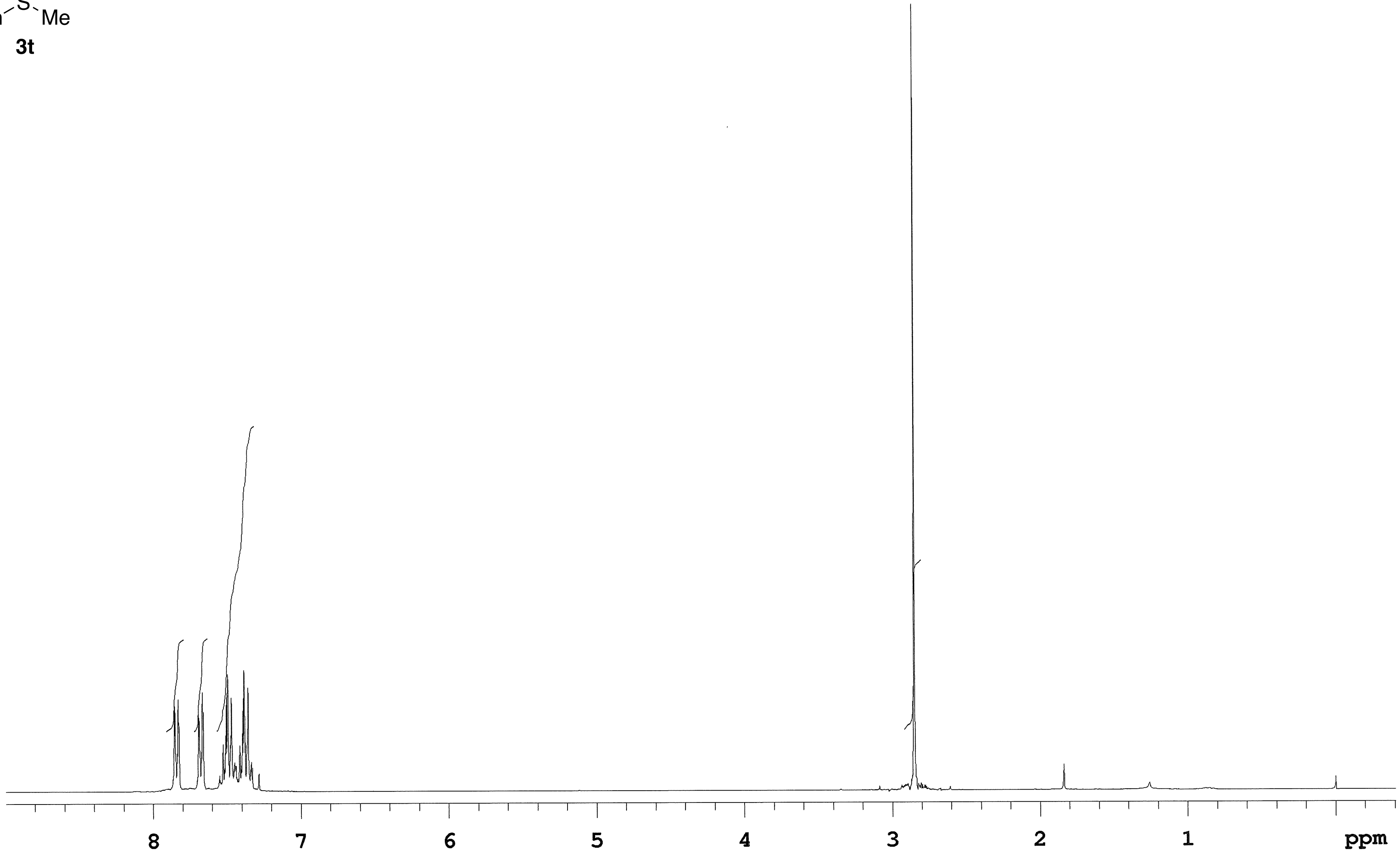

^1H NMR (500 MHz, $CDCl_3$)

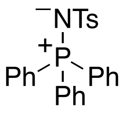

5

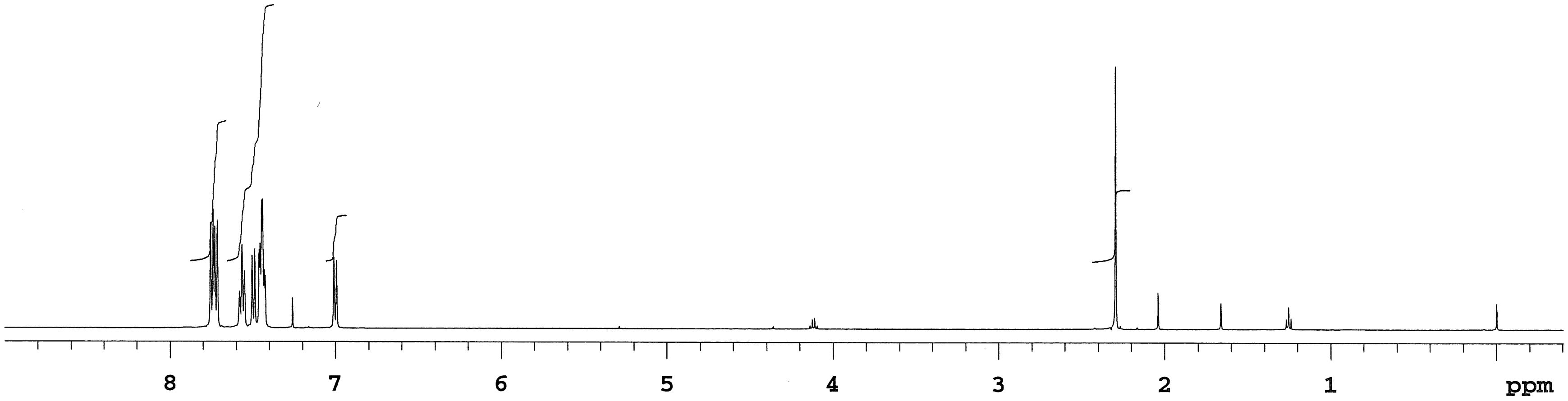

Supplement: Supplementary file 1 [file molecules-24-00979-s001.pdf]
